# Profilin binding couples chloride intracellular channel protein CLIC4 to RhoA–mDia2 signaling and filopodium formation

**DOI:** 10.1074/jbc.RA118.002779

**Published:** 2018-10-31

**Authors:** Elisabetta Argenzio, Jeffrey Klarenbeek, Katarzyna M. Kedziora, Leila Nahidiazar, Tadamoto Isogai, Anastassis Perrakis, Kees Jalink, Wouter H. Moolenaar, Metello Innocenti

**Affiliations:** From the Divisions of ‡ Cell Biology,; § Molecular Genetics, and; ¶ Biochemistry, The Netherlands Cancer Institute, Plesmanlaan 121, 1066 CX Amsterdam, The Netherlands

**Keywords:** membrane trafficking, G protein-coupled receptor, Rho (Rho GTPase), profilin, actin, G-actin, formin, epidermal growth factor (EGF), cell adhesion, cell motility, filopodia, lysophosphatidic acid

## Abstract

Chloride intracellular channel 4 (CLIC4) is a cytosolic protein implicated in diverse actin-based processes, including integrin trafficking, cell adhesion, and tubulogenesis. CLIC4 is rapidly recruited to the plasma membrane by RhoA-activating agonists and then partly colocalizes with β1 integrins. Agonist-induced CLIC4 translocation depends on actin polymerization and requires conserved residues that make up a putative binding groove. However, the mechanism and significance of CLIC4 trafficking have been elusive. Here, we show that RhoA activation by either lysophosphatidic acid (LPA) or epidermal growth factor is necessary and sufficient for CLIC4 translocation to the plasma membrane and involves regulation by the RhoA effector mDia2, a driver of actin polymerization and filopodium formation. We found that CLIC4 binds the G-actin–binding protein profilin-1 via the same residues that are required for CLIC4 trafficking. Consistently, shRNA-induced profilin-1 silencing impaired agonist-induced CLIC4 trafficking and the formation of mDia2-dependent filopodia. Conversely, CLIC4 knockdown increased filopodium formation in an integrin-dependent manner, a phenotype rescued by wild-type CLIC4 but not by the trafficking-incompetent mutant CLIC4(C35A). Furthermore, CLIC4 accelerated LPA-induced filopodium retraction. We conclude that through profilin-1 binding, CLIC4 functions in a RhoA–mDia2–regulated signaling network to integrate cortical actin assembly and membrane protrusion. We propose that agonist-induced CLIC4 translocation provides a feedback mechanism that counteracts formin-driven filopodium formation.

## Introduction

Chloride intracellular channel (CLIC)^3^ proteins (CLIC1–6) are small globular proteins (∼28 kDa) that are structurally related to the Omega-class GSH *S*-transferases, showing an N-terminal thioredoxin-like domain followed by an all α-helical C-terminal domain (1–4). However, CLICs have distinct but poorly understood cellular functions from the GSTs. Contrary to their original name, CLIC proteins do not function as conventional chloride channels but instead have been implicated in diverse actin-dependent processes, such as tubulogenesis, membrane remodeling, endosomal trafficking, vacuole formation, and cell adhesion, among others (3–5). It has recently been shown that CLICs have intrinsic glutaredoxin-like activity, at least under *in vitro* conditions with a conserved reactive cysteine serving as a key catalytic residue (6, 7), but whether CLIC glutaredoxin-like activity is maintained in the reducing cytosol is unknown.

CLIC4 is arguably one of the best-studied CLIC family members. Despite decades of research, progress in CLIC function has been frustratingly slow, partly because direct binding partners have been elusive. CLICs are often found associated with the cortical actin cytoskeleton and are detected on intracellular membranes, where they may participate in the formation and maintenance of vesicular compartments (5, 8–11). Growing evidence indicates that CLIC proteins play roles in actin-mediated trafficking events. CLIC4 knockout mice are viable but are smaller and show defects in actin-dependent processes, including delayed wound healing and impaired endothelial and epithelial tubulogenesis (12–14). Strikingly, CLIC4 undergoes rapid redistribution from the cytosol to the plasma membrane in response to G_12/13_-coupled receptor agonists, notably LPA (a major serum constituent) and other G protein–coupled receptor agonists (15, 16). CLIC4 translocation was strictly dependent on RhoA-mediated actin polymerization and, interestingly, on the reactive but enigmatic Cys-35 residue as well as on other conserved residues that in GSTs are critical for substrate binding (15). This strongly suggests that the substrate-binding features of the Omega GSTs have been conserved in the CLICs, along with the fold itself, and that binding of an as yet unknown partner (or substrate) is essential for CLIC4 function. Yet the putative binding partner and the functional relevance of agonist-induced CLIC4 trafficking have been elusive.

In epithelial cells, CLIC4 is homogeneously distributed and can colocalize with a subset of early and recycling endosomes (10). In response to serum or LPA stimulation, CLIC4 rapidly colocalizes with β1 integrins, consistent with CLIC4 functioning in actin-dependent exocytic–endocytic trafficking under the control of receptor agonists (15). A study on renal tubulogenesis confirmed that CLIC4 regulates intracellular trafficking, showing that CLIC4 colocalizes with the retromer complex and recycling endosomes, whereas CLIC4 depletion resulted in the enrichment of branched actin at early endosomes (13). Collectively, these findings establish CLIC4 as a trafficking regulator that acts in concert with the actin cytoskeleton.

A major challenge toward better understanding of the CLICs is the identification of specific binding partner(s); this should help to clarify how CLICs traffic to or associate with membrane compartments. In this study, we characterize CLIC4 trafficking and function in further mechanistic detail and establish the G-actin–binding protein profilin-1 as a direct interacting partner of CLIC4. Our results indicate that, through profilin-1 binding, CLIC4 functions in a RhoA–mDia2 and integrin-regulated signaling network to integrate cortical actin assembly and membrane protrusion.

## Results

### Rapid but transient translocation of CLIC4 to the plasma membrane induced by LPA and EGF

**Figure 1.**
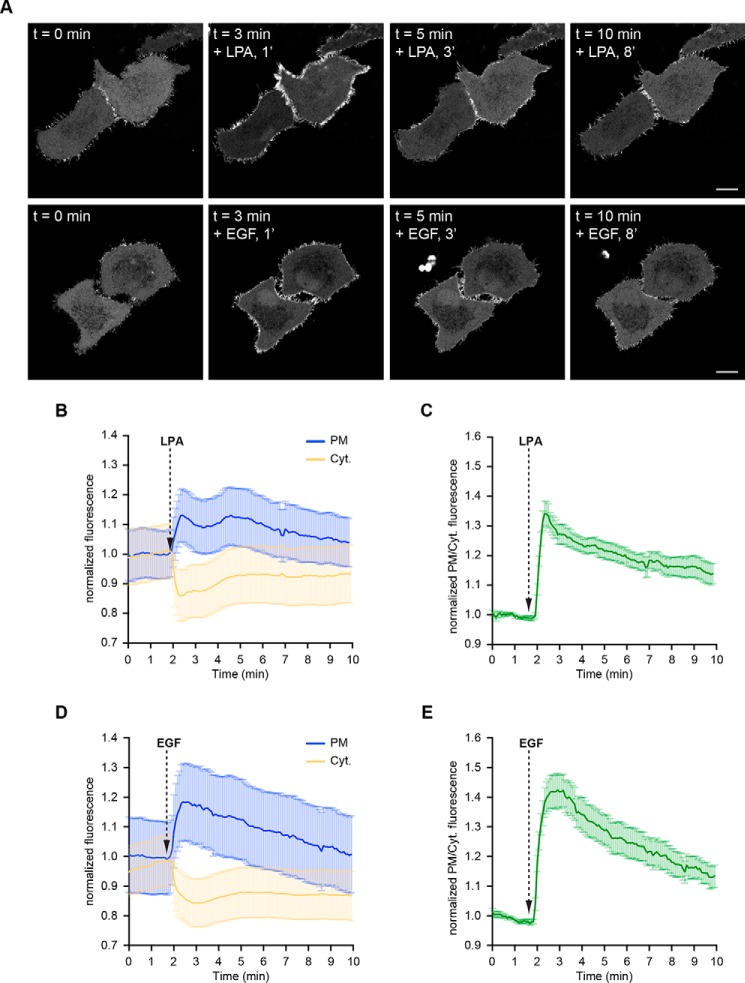
**LPA and EGF induce translocation of CLIC4 to the plasma membrane.** *A,* live-cell imaging of CLIC4 translocation to the plasma membrane. Cells were seeded on glass coverslips and transfected with YFP–CLIC4. LPA (2 μm, *top*) and EGF (100 ng/ml) were added 2 min after starting imaging. Frames from time-lapse movies at the indicated time points are shown. *Scale bar,* 10 μm. *B–E,* quantification of LPA- and EGF-induced CLIC4 translocation. *B* and *D,* translocation was quantified by measuring YFP fluorescence at the plasma membrane (*PM, blue line*) and in cytoplasm (*Cyt., yellow line*). Mean ± S.E. of normalized plasma membrane and cytosolic CLIC4 fluorescence are plotted over time (LPA, *n* = 16 cells; EGF *n* = 18 cells, from two independent experiments). *C* and *E,* net translocation is expressed as mean ± S.E. of the normalized PM/Cyt. fluorescence ratio (LPA, *n* = 16 cells; EGF *n* = 18 cells, from two independent experiments).

In serum-deprived neuronal and epithelial cells, CLIC4 resides mainly in the cytosol, where it is highly mobile (15), and to a lower extent in distinct patches at the plasma membrane. Using HeLa cells, we found that CLIC4 is rapidly recruited to the plasma membrane not only by G_12/13_–RhoA-coupled receptor agonists such as lysophosphatidic acid (LPA) but also, somewhat unexpectedly, by a prototypic receptor tyrosine kinase ligand, notably epidermal growth factor (EGF) (Fig. 1*A* and supporting Movies S1 and S2). Receptor-mediated CLIC4 accumulation at the plasma membrane coincided with CLIC4 depletion from the cytosol (Fig. 1, *B* and *D*), occurring within seconds of receptor stimulation. Maximum membrane recruitment was reached within ∼1 min of LPA or EGF addition. Subsequently, CLIC4 gradually disappeared from the plasma membrane as it translocated back to the cytosol over a time course of about 10 min in the continuous presence of agonist (Fig. 1, *B–E*). Similar to LPA, EGF induced CLIC4 to translocate to β1 integrins at the plasma membrane (Fig. S1*A*) (10). CLIC4 translocation induced by either LPA or EGF was blocked by the G-actin–binding toxin latrunculin A, indicating that CLIC4 trafficking depends on F-actin polymerization (15). Furthermore, using an EGFR inhibitor, we ruled out that LPA acts through EGFR transactivation (Fig. S1*B*). It thus appears that EGF and G_12/13_-coupled receptors share a common signaling mechanism to evoke CLIC4 trafficking.

CLIC4 shows oxidoreductase activity toward artificial substrates *in vitro*, with Cys-35 serving as active-site residue, which is inhibited by ethacrynic acid and IAA-94, compounds once used as chloride channel blockers (6). Because CLIC4 trafficking depends on residue Cys-35 (15), we pretreated the cells with these membrane-permeable inhibitors but found no effect on agonist-induced CLIC4 trafficking (Fig. S2). These results argue against intrinsic oxidoreductase activity playing a role in CLIC4 trafficking.

### RhoA activation is necessary and sufficient to drive CLIC4 translocation

**Figure 2.**
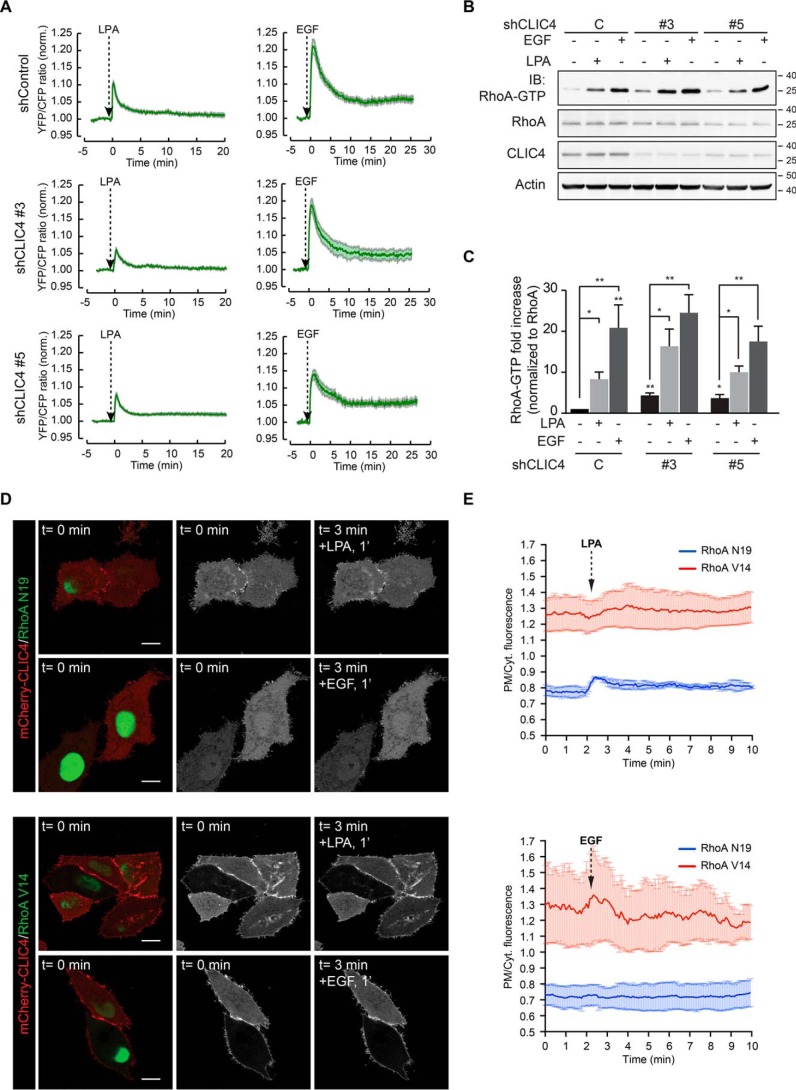
**LPA- and EGF-induced translocation of CLIC4 depends on RhoA activation.** *A,* kinetics of RhoA activation by LPA and EGF and dependence on CLIC4. shControl and shCLIC4 knockdown cells were transfected with a RhoA biosensor (17). RhoA activity is plotted as normalized YFP/CFP ratio over time (LPA: shControl = 10 cells, shCLIC4 #3 = 15 cells, and shCLIC4 #5 = 15 cells, from at least two independent experiments; EGF: shControl = 15 cells, shCLIC4 #3 = 15 cells, and shCLIC4 #5 = 10 cells, from at least two independent experiments). *B* and *C,* RhoA pulldown assays. shControl and shCLIC4 knockdown HeLa cells were serum-starved overnight and either stimulated with LPA (2 μm) or EGF (100 ng/ml) for 3 min or left untreated. GTP-bound RhoA was pulled down as described under “Experimental procedures.” GTP-bound and total RhoA were detected by immunoblot analysis using anti-RhoA antibodies. CLIC4 knockdown was monitored by immunoblot analysis of total cell lysates using anti-CLIC4 antibodies. Actin was used as loading control. Representative blots of one out of six independent experiments are shown. Densitometric analysis (mean ± S.E.) of six experiments is shown in *C* along with the results of one-way ANOVA with Dunnett's multiple comparisons test (*, *p* < 0.05; **, *p* < 0.01). *D,* HeLa cells were plated on glass coverslips and cotransfected with mCherry–CLIC4 and either dominant-negative (RhoAN19, *top*) or constitutively active RhoA (RhoAV14, *bottom*) in a bicistronic IRES vector expressing GFP. Cells were serum-starved and either stimulated with LPA (2 μm) or EGF (100 ng/ml). RhoAN19- and RhoAV14-transfected cells express monomeric GFP. Frames from time-lapse movies at the indicated time points are shown. *Scale bars,* 10 μm. *E,* quantification of agonist-induced CLIC4 translocation in RhoAN19-expressing (*blue trace*) and RhoAV14-expressing (*red trace*) cells. LPA-induced (*top*) and EGF-induced (*bottom*) net translocation are expressed as mean ± S.E. of the PM/Cyt. fluorescence ratio.

Given the apparent involvement of RhoA in CLIC4 translocation (15), we set out to monitor RhoA activation in real time in LPA- and EGF-stimulated cells, as well as in CLIC4-depleted cells, using a RhoA-specific FRET-based biosensor (17). As shown in Fig. 2*A*, both LPA and EGF rapidly activated RhoA in HeLa cells. Somewhat unexpectedly, EGF was a stronger RhoA activator than LPA in these cells. Peak activation of RhoA by either LPA or EGF was reached within 1 min; thereafter, Rho-GTP levels gradually decreased over a 10-min time period, stabilizing at a level above pre-stimulation values. CLIC4 knockdown (using two independent shRNAs) consistently led to a small increase in basal RhoA-GTP levels as revealed in pulldown assays (Fig. 2, *B* and *C*), possibly secondary to altered integrin function. However, CLIC4 depletion did not affect agonist-induced RhoA activation, neither in amplitude nor in kinetics (Fig. 2, *A* and *B*). It is of note that the RhoA activation kinetics closely parallels the time course of CLIC4 translocation (Fig. 2*A*), consistent with RhoA activity providing the driving force for CLIC4 recruitment to the plasma membrane.

Indeed, expression of dominant-negative RhoA(N19) blocked agonist-induced CLIC4 translocation (Fig. 2*D*), although constitutively active RhoA(V14A) forced CLIC4 to accumulate permanently at the plasma membrane (Fig. 2*D*). Under the latter conditions, translocation of the remaining cytosolic CLIC4 could not be further stimulated by LPA (Fig. 2, *D* and *E*). Thus, RhoA activation is a necessary and sufficient signal for CLIC4 to translocate. Moreover, these results imply that agonist-induced CLIC4 translocation to the plasma membrane may serve as a convenient readout of RhoA activation.

### Involvement of Rho effector mDia2

Major downstream effectors of RhoA are Rho-kinase (ROCK) and the mDia formins. ROCK induces actomyosin-mediated cell contraction, whereas the formins mDia1 and mDia2 promote Rho GTPase–regulated nucleation and elongation of linear actin filaments to drive cell protrusion. ROCK inhibition did not affect CLIC4 translocation (Fig. S3) (15). We therefore focused our attention on mDia1 and mDia2, which are the two major formins in HeLa cells (18).

**Figure 3.**
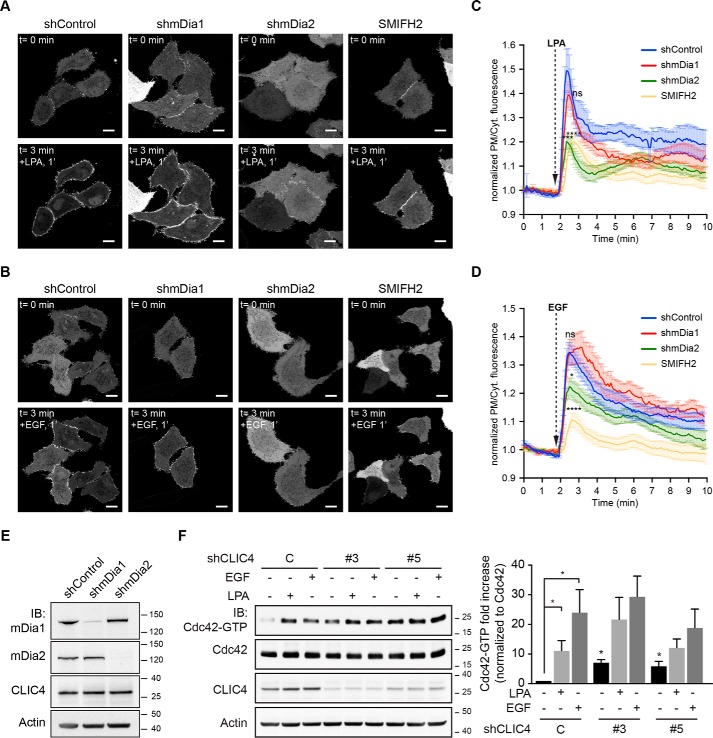
**LPA-induced translocation of CLIC4 relies on mDia2 and its activity.** *A* and *B,* live-cell imaging of CLIC4 translocation in mDia knockdown HeLa cells. Stable mDia1 and mDia2 knockdown HeLa cells were obtained as described under “Experimental procedures.” shControl, shmDia1 (#2 (18)), and shmDia2 (#1 (44)) cells were seeded on glass coverslips and transfected with YFP–CLIC4. shControl cells were pre-treated with SMIFH2 (50 μm, 20 min) or left untreated. LPA (*A*) (2 μm) or EGF (*B*) (100 ng/ml) were added 2 min after starting imaging. SMIFH2 was maintained during stimulation. Frames from time-lapse movies at the indicated time points are shown. *Scale bar,* 10 μm. *C,* quantification of LPA-induced translocation in shControl (*blue trace, n* = 8 cells), shmDia1 (*red trace, n* = 8 cells), shmDia2 (*green trace, n* = 12 cells), and SMIFH2-treated cells (*yellow trace, n* = 12 cells) from two independent experiments. One-way ANOVA with Dunnett's multiple comparisons test was performed comparing the highest values of the curves (***, *p* < 0.001; ****, *p* < 0.0001, *ns,* non-significant). *D,* quantification of EGF-induced translocation in shControl (*blue trace, n* = 16 cells), shmDia1 (*red trace, n* = 9 cells), shmDia2 (*green trace, n* = 12 cells), and SMIFH2-treated cells (*yellow trace, n* = 12 cells), from two independent experiments. One-way ANOVA with Dunnett's multiple comparisons test was performed comparing the highest values of the curves (*, *p* < 0.05; ****, *p* < 0.0001, *ns*, nonsignificant). Net translocation in *C* and *D* is expressed as mean ± S.E. of the normalized PM/Cyt. ratio obtained from the analyzed cells. *E,* knockdown validation was achieved by immunoblotting (*IB*) using anti-mDia1 and anti-mDia2 antibodies (see also Fig. S4C). Actin was used as loading control. *F,* Cdc42 pulldown assays. shControl and shCLIC4 knockdown HeLa cells were serum-starved overnight and either stimulated with LPA (2 μm) or EGF (100 ng/ml) for 3 min or left untreated. GTP-bound Cdc42 was pulled down as described under “Experimental procedures.” GTP-bound and total Cdc42 were detected by immunoblot analysis using anti Cdc42 antibody. *Left*, representative blots of one out of three independent experiments are shown. *Right*, *bar graph* shows normalized Cdc42–GTP levels as mean ± S.E. of three independent experiments (one-way ANOVA with Dunnett's multiple comparisons test, *, *p* < 0.05).

Stable knockdown of either mDia1 or mDia2 (using lentiviral vectors shmDia1 and shmDia2, respectively) did not alter the subcellular localization of CLIC4 (Fig. 3, *A* and *C*) nor its total expression level (Fig. 3*E*). Although CLIC4 translocation was not affected by mDia1 knockdown, it was prominently reduced upon mDia2 depletion (Fig. 3, *A–D*; second independent hairpin shown Fig. S4). Moreover, the pan-formin inhibitor SMIFH2 (19, 20) caused a similar decrease in CLIC4 translocation (Fig. 3, *A–D*). Thus, translocation of CLIC4 is in large part regulated by the RhoA–mDia2 signaling axis through cortical actin polymerization. However, we found no interaction between recombinant CLIC4 and purified F-actin in cosedimentation experiments (see Fig. 6*A*, below). Because CLIC4 recruitment was not fully inhibited upon mDia2 knockdown, it is noteworthy that both LPA and EGF also induced activation of Rho–GTPase family member Cdc42 (Fig. 3*F*). However, LPA activates Cdc42 through G_i_ signaling (17), whereas CLIC4 trafficking is independent of G_i_ (15). We therefore conclude that agonist-induced CLIC4 translocation is regulated, at least in large part, by RhoA–mDia2–driven actin polymerization at the cell periphery.

### CLIC4 binds profilin-1 via conserved residues, including Cys-35

**Figure 4.**
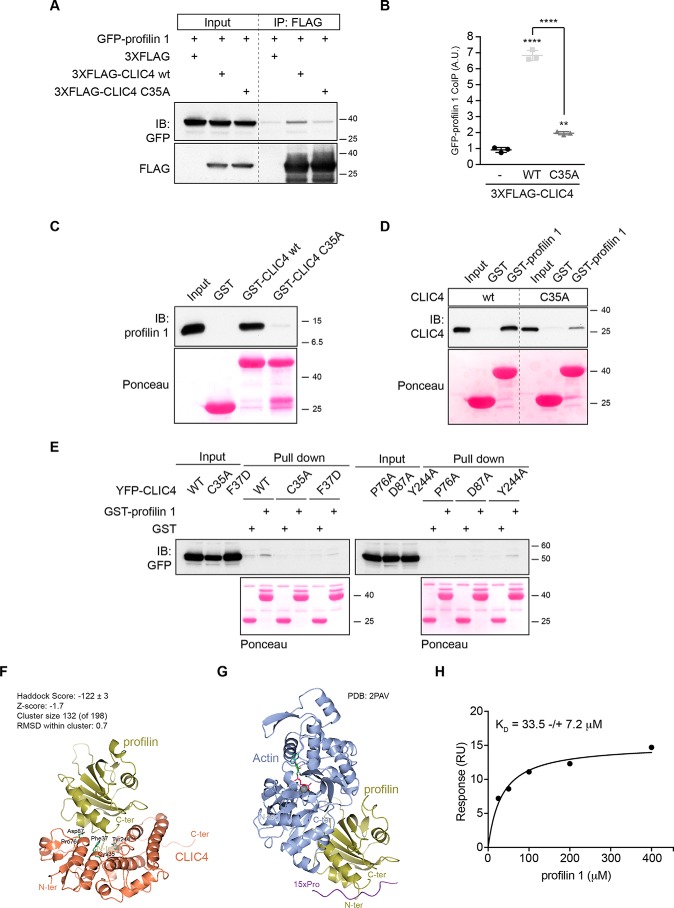
**CLIC4 binds profilin-1 via distinct residues that make up an open cleft.** *A,* profilin-1 coimmunoprecipitates (*IP*) with CLIC4 in cells. 3×FLAG-CLIC4 WT (*wt*) and its mutant in Cys-35 (C35A) were cotransfected with GFP-profilin-1 in HEK293 cells. CLIC4 was immunoprecipitated from cell lysate (1 mg) using anti FLAG antibodies. Coimmunoprecipitated profilin-1 and CLIC4 were detected by immunoblotting (*IB*) using anti-GFP and anti-FLAG antibodies, respectively. Representative blots of one out of three independent experiments are shown. *B,* densitometric analysis shows coimmunoprecipitated GFP–profilin-1 (mean ± S.D.) from three independent experiments (one-way ANOVA with Tukey's multiple comparisons test, **, *p* < 0.01; ****, *p* < 0.0001). *C* and *D,* CLIC4 directly binds profilin-1 *in vitro. C,* purified immobilized GST–CLIC4 WT and GST–CLIC4 C35A mutant were incubated with profilin-1 for 2 h on ice and pulled down with GST–agarose beads. The amount of profilin-1 pulled down by GST–CLIC4 was detected by immunoblotting using anti-profilin-1 antibodies. The reciprocal experiment was performed using GST–profilin-1 and CLIC4 WT and the C35A mutant (*D*). GST alone was used as a control. Ponceau staining showed equal loading of the GST-fusion proteins. Representative blots of one out of three independent experiments are shown. *E,* YFP–CLIC4 (WT) and the indicated mutants were transfected into HEK293 cells. Total-cell lysate (1 mg) was incubated with GST or GST–profilin-1. The amount of YFP–CLIC4 WT and mutants pulled down by profilin-1 was detected by immunoblot analysis using anti-GFP antibodies. Ponceau staining showed equal loading of the GST-fusion proteins. Representative blots of one out of two independent experiments are shown. *F* and *G,* molecular modeling of profilin-1 interactions. *F,* HADDOCK computational model showing CLIC4 (*orange*) and profilin-1 (*gold*). The CLIC4 residues discussed under “Results,” and the N and C termini of both proteins are indicated. *G,* crystal structure of profilin-1 (*gold*) binding to G-actin (*blue*) (PDB code 2PAV). See text for further details. *H,* equilibrium dissociation constant of the CLIC4–profilin-1 is 33.5 ± 7.2 μm. *Graph* shows steady-state response measured by SPR (*RU* = resonance units) at the indicated profilin-1 concentrations. The equilibrium dissociation constant (*K_D_*) is expressed as mean ± S.D., and the coefficient of determination of the fitting (*R*^2^) is 0.95.

Because CLIC4 translocation depends on mDia2 activity, we focused our attention on the mDia2-interacting protein profilin-1 (encoded by *PFN1*), which modulates the activity of formins (21–23). Profilin-1 is a ubiquitous G-actin–binding protein with separate binding sites for actin and poly-Pro stretches, such as those found in the FH1 domains of formins (22, 23). We examined a possible interaction, both biochemical and functional, between CLIC4 and profilin-1. As shown in Fig. 4, *A* and *B*, profilin-1 coimmunoprecipitated with CLIC4 in transfected HEK293 cells. However, the translocation-incompetent CLIC4(C35A) mutant showed a markedly reduced interaction with profilin-1 (Fig. 4, *A* and *B*). We established that the interaction is direct by using recombinant purified CLIC4 and profilin-1 in pulldown assays (Fig. 4, *C* and *D*). Remarkably, binding of profilin-1 was impaired not only in CLIC4(C35A) but also in other CLIC4 mutants that fail to translocate (15), notably the F37D, P76A, D97A, and Y244A mutants (Fig. 4*E*). These respective residues are highly conserved and lie in a concave surface (or “cleft”) of CLIC4, similar to that observed in the Omega GSTs where it mediates GSH binding (1, 15). However, CLIC proteins exhibit only very-low affinity for GSH. Yet, it is conceivable that CLICs might use this cleft as a binding site for an extended macromolecular chain, notably a polypeptide or a post-translationally modified protein, to be targeted to a particular subcellular location (1, 3, 15, 24, 25).

To gain insight into the CLIC4-binding site of profilin-1, we examined whether CLIC4 may regulate the actin-binding properties of profilin-1. To this end, we exploited that profilin-1 inhibits actin self-assembly by forming a 1:1 complex with G-actin. We found no effect of recombinant CLIC4 on the kinetics of profilin-1-actin polymerization measured *in vitro* (see Fig. 6*B*, below), showing that CLIC4 and G-actin do not compete for profilin-1. Moreover, CLIC4 did not affect spontaneous actin polymerization (see Fig. 6*C*, below). Together, these results show that CLIC4 in itself has no direct effects on actin dynamics and suggest that the CLIC4- and G-actin–binding surfaces of profilin-1 are distinct.

### Molecular modeling of the CLIC4–profilin-1 complex

We set out to create a model of the CLIC4–profilin-1 complex using the High ambiguity Driven protein–protein DOCKing (HADDOCK) modeling suite (26, 27). HADDOCK modeling resulted in a large cluster (132 models, 67% of total) of similar models (0.7 Å RMSD from the overall lowest-energy structure) with excellent scores (Fig. 4*F*). The CLIC4 interaction surface is located on the other end of the profilin-1–binding site to G-actin and is similar to the binding region of poly-proline (poly-Pro) peptides (Fig. 4*G*) (28, 29). Thus, the modeling results corroborate our biochemical observations that CLIC4 and G-actin do not compete for profilin-1 with a plausible structural explanation.

Using surface plasmon resonance (SPR) to determine the equilibrium dissociation constant of the CLIC4–profilin-1 complex, we found saturable binding with a *K_D_* of 33.5 μm (Fig. 4*H*). This relatively low binding affinity may not come as a surprise because physiologically relevant interactions mediated by the poly-Pro–binding site of profilin-1 are in a similar range (28, 30). Furthermore, weak interactions may allow CLIC proteins to transiently interact with distinct binding partners along a given trafficking route.

In any case, these results establish profilin-1 as a direct binding partner of CLIC4, and they link profilin-1 binding to the capability of CLIC4 to respond to RhoA activation.

### Involvement of profilin-1 in LPA- and EGF-induced CLIC4 translocation

**Figure 5.**
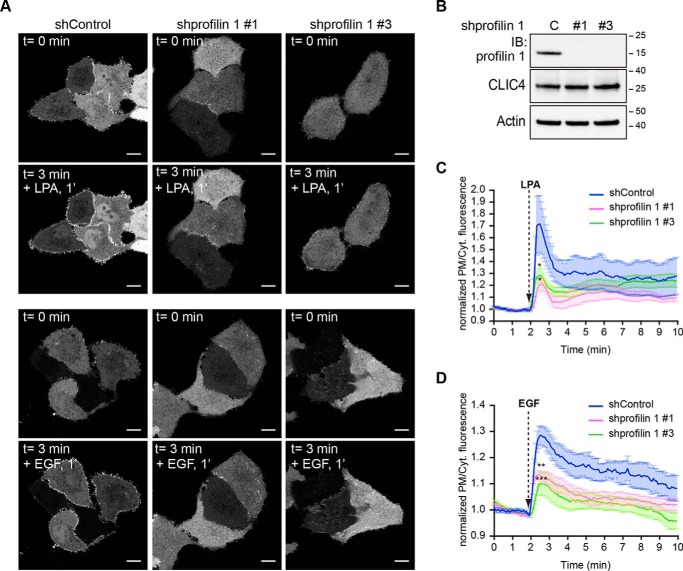
**Role of profilin-1 in LPA-induced CLIC4 translocation.** *A,* live-cell imaging of CLIC4 translocation in profilin-1 knockdown HeLa cells. Stable profilin-1 knockdown cells were obtained as described under “Experimental procedures” using two distinct *PFN1*-targeting shRNAs. Cells were seeded on glass coverslips and transfected with YFP–CLIC4. LPA (2 μm) and EGF (100 ng/ml) were added 2 min after starting imaging. Frames from time-lapse movies at the indicated time points are shown. *Scale bar,* 10 μm. *B,* characterization of profilin-1 knockdown HeLa cells. Total-cell lysates obtained from shControl (*C*), shprofilin-1 #1 and shprofilin-1 #3 (*#1* and *#3*) knockdown cells were immunoblotted (*IB*) with profilin-1 antibodies; actin was used as loading control. Hairpin #1 and #3 reduced profilin-1 protein levels by 95 ± 2 and 92 ± 4% (mean ± S.E., *n* = 3). RT-qPCR was used to independently validate the level of *PFN1* knockdown (relative mRNA expression of *PFN1* (mean ± S.D., *n* = 3): shControl = 1 ± 0.092; shprofilin-1 #1 = 0.038 ± 0.149; shprofilin-1 #3 = 0.075 ± 0.084). *C,* quantification of LPA-induced CLIC4 translocation (*n*_shControl_ = 7 cells; *n*_shprofilin-1 #1_ = 6 cells; *n*_shprofilin-1 #3_ = 12 cells, from two independent experiments). One-way ANOVA with Dunnett's multiple comparisons test was performed comparing the highest values of the curves (*, *p* < 0.05). *D,* quantification of EGF-induced CLIC4 translocation (*n*_shControl_ = 11; *n*_shprofilin-1 #1_ = 14; *n*_shprofilin-1 #3_ = 11 cells, from two independent experiments). One-way ANOVA with Dunnett's multiple comparisons test was performed comparing the highest values of the curves, **, *p* < 0.01; ***, *p* < 0.001. Net translocation in *C* and *D* is expressed as mean ± S.E. of the normalized PM/Cyt. ratio obtained from the analyzed cells.

To examine the importance of profilin-1 in CLIC4 trafficking, we generated profilin-1–depleted HeLa cells using two distinct shRNAs; these cells showed unaltered CLIC4 expression and localization (Fig. 5, *A* and *B*). LPA- and EGF-induced CLIC4 translocation to the plasma membrane was strongly reduced upon profilin-1 depletion (Fig. 5, *B* and *C*). Furthermore, the filopodia induced by mDia2 were decreased in these cells (Fig. S5). These results support the view that profilin-1 binding connects CLIC4 to RhoA–mDia2 regulated actin dynamics.

### CLIC4 does not affect actin nucleation by mDia2 in vitro

**Figure 6.**
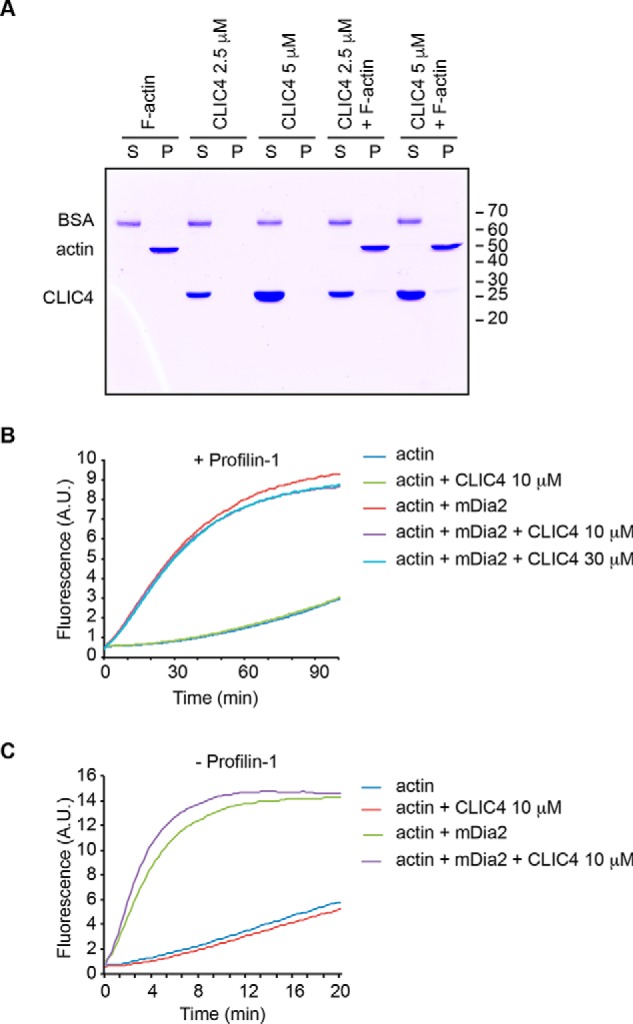
**CLIC4 does not bind F-actin and has no effect on actin polymerization.** *A,* CLIC4 does not bind F-actin *in vitro*. Cosedimentation assays were performed mixing recombinant purified full-length CLIC4 (2.5 and 5 μm) with BSA (0.3 μm) and either F-actin (2.5 μm) or F-actin buffer as described under “Experimental procedures.” The same percentages of soluble (*S*) and pelleted (*P*) fractions were subjected to SDS-PAGE followed by Coomassie Brilliant Blue staining. Data are representative of three independent experiments performed using three different actin and CLIC4 preparations. *B* and *C,* CLIC4 does not affect spontaneous actin polymerization or mDia2-mediated actin nucleation *in vitro*. Bulk actin polymerization was assayed as described under “Experimental procedures” using purified recombinant CLIC4 (10 and 30 μm in *B*) 10 μm in *C*, actin (2 μm in *B*, 1 μm in *C*), mDia2-FH1–FH2 (0.1 μm), in the presence (+) or absence (−) of profilin-1 (5 μm) as indicated.

The similarities between the poly-Pro- and CLIC4–binding sites of profilin-1 suggest that CLIC4 may regulate mDia2-driven actin dynamics. Because profilin-1 accelerates formin-mediated actin assembly (31, 32), we measured actin polymerization induced by the FH1–FH2 domain of mDia2 in the presence of profilin-1 with or without recombinant CLIC4. mDia2 increased the rate of actin polymerization, as expected, but this was not affected by CLIC4 (Fig. 6*B*). We ruled out that CLIC4 interferes directly with mDia2 by repeating these assays without profilin-1 (Fig. 6*C*). Thus, CLIC4 does not affect actin nucleation by mDia2 *in vitro* when actin polymerization occurs in solution. Nevertheless, it remains possible that CLIC4 regulates mDia2 polymerase activity (31–33). Because the affinities of CLIC4 and mDia2 for profilin-1 are both in the high micromolar range, CLIC4 may reduce binding or activation of mDia2 by profilin-1 at membranes where bi-dimensionality increases their local concentration.

### CLIC4 depletion promotes integrin-dependent filopodium protrusion

**Figure 7.**
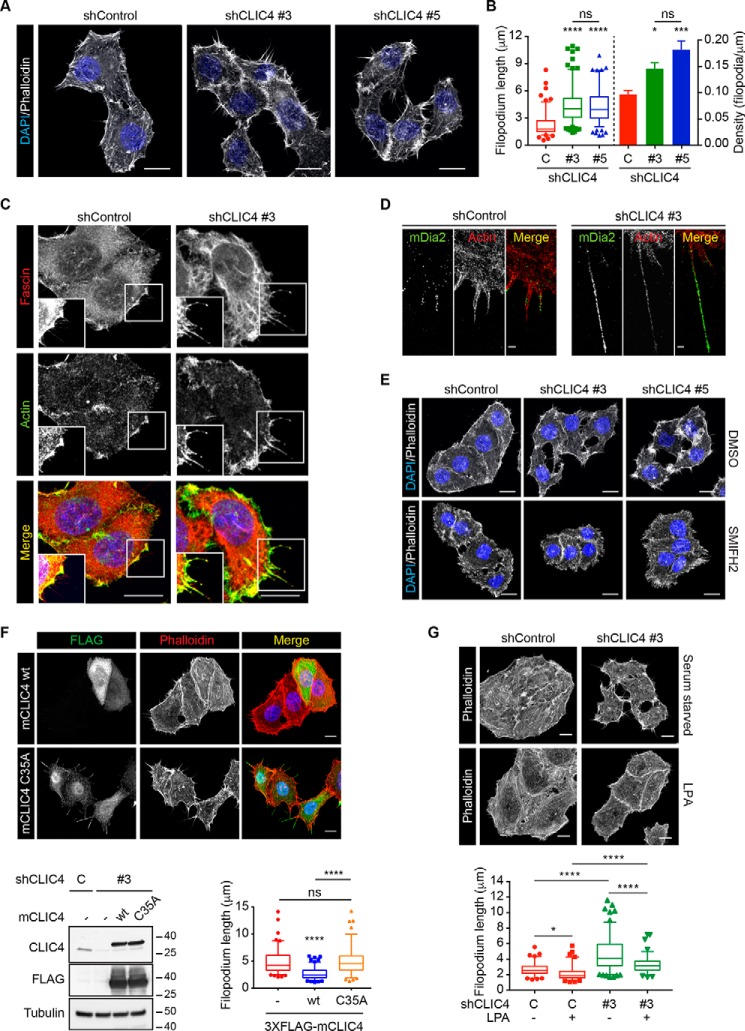
**CLIC4 depletion increases filopodium formation.** *A,* effect of CLIC4 depletion on filopodia. Control and CLIC4 knockdown HeLa cells were seeded on collagen-I–coated glass coverslips, serum-starved overnight, and fixed. Maximal projections of confocal Z-stacks show actin cytoskeleton and nuclear stained with phalloidin (*gray*) and DAPI (*blue*), respectively. *Scale bar,* 10 μm. *B,* quantification of filopodium length and density. Data represent filopodium length and density measured in two independent experiments as described under “Experimental procedures” (*n*_shControl_ = 28 cells (9 images), *n*_shCLIC4 #3_ = 34 cells (10 images), and *n*_shCLIC4 #5_ = 31 (10 images)). *Filopodial length*: *box* represents the 25th to 75th percentiles with *line and whiskers* indicating the median and the 5th and the 95th percentiles, respectively. *Bar graph* depicts filopodial density as mean ± S.E. One-way ANOVA with Tukey's multiple comparisons test (*, *p* < 0.05; ***, *p* < 0.001; ****, *p* < 0.0001, *ns*, nonsignificant). Note that all cells formed filopodia. *C,* fascin staining. Cells were treated as in *A*. Confocal images of cells stained with actin (*green*), fascin (*red*), and DAPI (*blue*) are shown. *Insets* show 1.31-fold magnifications of *boxed areas* in the main image. *Scale bar,* 10 μm. *D,* super-resolution imaging of mDia2 in filopodia. Cells were seeded on collagen-I–coated glass coverslips, transfected with FLAG-mDia2 WT, and serum-starved overnight. Super-resolution images of mDia2 WT (*green*) and phalloidin (actin, *red*). *Scale bar,* 500 nm. *E,* SMIFH2 treatment reduces filopodium length. Cells were seeded on collagen-I–coated glass coverslips, serum-starved overnight, and incubated with SMIFH2 (50 μm, 20 min) or mock-treated (DMSO) before fixation. Maximal projections of confocal Z-stacks show actin cytoskeleton and nuclei stained with phalloidin (*gray*) and DAPI (*blue*), respectively. *Scale bar,* 10 μm. *F,* rescue of filopodium length in CLIC4 knockdown cells using CLIC4 WT and CLIC4 C35A. CLIC4 knockdown HeLa cells were seeded on collagen-I–coated glass coverslips and transfected with either WT 3×FLAG-mCLIC4 (mCLIC4 WT) or 3×FLAG-mCLIC4 C35A (mCLIC4(C35A)) as *Mus musculus* CLIC4 is insensitive to the employed shRNAs. Cells were serum-starved overnight and fixed. *Top,* maximal projections of confocal Z-stacks show CLIC4, actin cytoskeleton, and nuclei stained with anti-FLAG antibody (*green*), phalloidin (*gray*), and DAPI (*blue*), respectively. Note rescue of both low and high CLIC4 expressors. *Scale bar,* 10 μm. *Bottom left,* representative blot showing the expression levels of WT (*wt*) and C35A 3×FLAG–mCLIC4. *Bottom right,* data representing filopodium length measured in two independent experiments were plotted and analyzed as in *B* (*n*_nontransfected (−)_ = 25 cells, *n*_3×FLAG-mCLIC4 WT_ = 26 cells, *n*_3×FLAG-mCLIC4(C35A)_ = 25 cells; ****, *p* < 0.0001, *ns,* nonsignificant). *G,* effect of LPA stimulation on filopodium length. shControl and shCLIC4 cells were seeded on collagen-I–coated coverslips and serum-starved overnight. Cells were stimulated with LPA (2 μm, 2 min) or left untreated before fixation. Maximal projections of confocal Z-stacks stained with phalloidin (*gray*) are shown. *Scale bar,* 10 μm. Data representing filopodium length measured in two independent experiments were plotted and analyzed as in *B* (*n*_shControl starvation_ = 25 cells, *n*_shControl LPA_ = 24 cells, *n*_shCLIC4 #3 starvation_ = 30 cells, *n*_shCLIC4 #3 LPA_ = 22 cells; *, *p* < 0.05; ****, *p* < 0.0001).

In addition to altering integrin trafficking (10), we found that CLIC4 depletion triggered the formation of long filopodium-like protrusions (Fig. 7, *A* and *B*). These protrusions were positive for the F-actin–bundling protein Fascin (Fig. 7*C*) and showed mDia2 at the tips (Fig. 7*D* and Fig. S6). Filopodium formation is regulated by formins and can be induced by Cdc42 GTPase activity (34–36). Consistent with this, Cdc42 activity was up-regulated upon CLIC4 knockdown (Fig. 3*F*), and the formin inhibitor SMIFH2 caused these protrusions to disappear (Fig. 7*E*). The observed finger-like protrusions therefore qualify as genuine filopodia. Given the effects of CLIC4 knockdown on integrin signaling and cell adhesion (10), these filopodia may reflect an integrin-dependent phenotype. Indeed, filopodia were similar when control and CLIC4 knockdown cells were grown on poly-lysine (Fig. S7). As CLIC4 regulated filopodial length and number only in cells plated on fibronectin, these results imply regulation by integrin-dependent signaling pathways.

Importantly, this CLIC4 knockdown phenotype could be rescued by reintroducing shRNA-resistant mouse CLIC4 (3×FLAG-mCLIC4) (Fig. 7*F*), whereas the profilin-1-binding–incompetent and trafficking-incompetent CLIC4(C35A) mutant could not. These findings underscore the importance of profilin-1 binding in dictating the CLIC4-regulated phenotype. Finally, LPA treatment reduced filopodium length in both control and CLIC4-depleted cells (Fig. 7*G*). This is consistent with LPA promoting actomyosin tension, which has a net negative effect on formins (33) and boosts the actin retrograde flow (37, 38). Yet, filopodia retraction by LPA was less complete upon CLIC4 depletion (Fig. 7*G*), unveiling the involvement of CLIC4 in this process.

**Figure 8.**
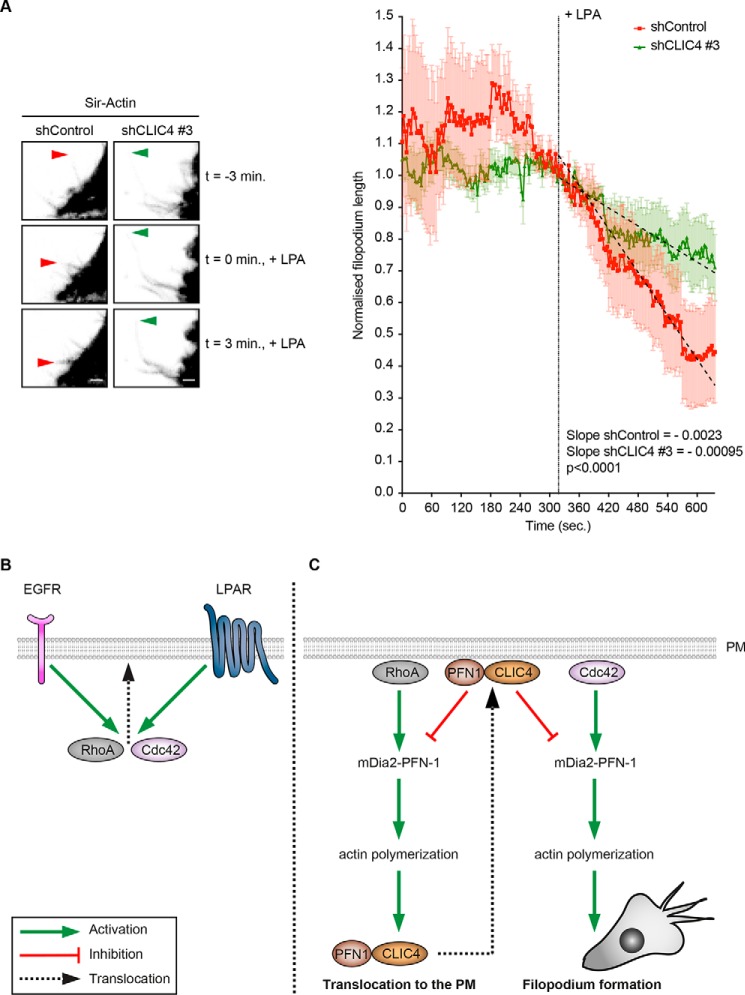
**CLIC4 translocation controls the filopodium retraction rate.** *A,* CLIC4 depletion reduces the filopodium retraction rate in LPA-stimulated cells. Control (*shControl*) and CLIC4 knockdown (*shCLIC4 #3*) HeLa cells were seeded on collagen-I–coated glass-bottom Petri dishes and serum starved overnight. Subsequently, cells were labeled with Sir-Actin, and filopodia were tracked as described under “Experimental procedures.” *Left,* frames taken from Movies 3 and 4 show selected filopodia in control and CLIC4 knockdown cells, respectively, at the indicated times before and after LPA stimulation. *Arrowheads* mark the tips of filopodia that were tracked and quantified. *Scale bar,* 2 μm. *Right,* length of 10 different filopodia obtained from control (*red trace*) and CLIC4 knockdown (*green trace*) cells (8 and 7 cells, respectively, from two independent experiments) was normalized and plotted against time (*sec.* = seconds) as mean ± S.E. Segmental linear regression was used to fit the retractions traces (*dashed black lines*) and calculate the slopes. *B* and *C,* proposed signaling scheme of how agonist-induced CLIC4 translocation regulates filopodium formation. *B,* EGF and LPA activate RhoA and Cdc42, leading to their translocation to the plasma membrane (*PM*). *C,* activated RhoA signals through mDia2 to promote actin polymerization in a profilin-1 (*PFN-1*)-regulated manner. This triggers rapid CLIC4 translocation to the plasma membrane, a process that requires CLIC4 binding to profilin-1 (PFN-1). At the plasma membrane, CLIC4 counteracts mDia2 downstream of both RhoA and Cdc42 and thereby modulates CLIC4 translocation and filopodium formation in a negative feedback loop.

To understand how CLIC4 translocation regulates filopodium length, we tracked filopodia in control and CLIC4-depleted cells before and after LPA stimulation (supporting Movies S3 and S4). Basal filopodial dynamics were asynchronous and characterized by extension and retraction phases of variable rate and duration (Fig. S8). Instead, filopodia synchronized on LPA addition and switched to a more coherent retraction phase (Fig. 8*A* and Fig. S8). The retraction rate was two times higher in the control than in the CLIC4 KD cells (Fig. 8*A*), and length variations were primarily due to changes in the filopodium tip position (supporting Movies S3 and S4). Notably, CLIC4 down-regulation also increased filopodial density (Fig. 7*B*), suggesting that CLIC4 may inhibit actin polymerization by formins at the plasma membrane. Given that the activities of Cdc42 (Fig. 3*C*) and RhoA (Fig. 2, *A–C*), key regulators of actomyosin-based tension and F-actin retrograde flow (37–39), are independent of CLIC4 in LPA-treated cells, we conclude that CLIC4 primarily controls formins at the tip of filopodia.

Collectively, our findings suggest that recruitment of CLIC4 to the plasma membrane serves to suppress the formation of integrin-regulated filopodia, along with promoting integrin trafficking (10).

## Discussion

This study sheds new light on the actin-based functions of CLIC4, in particular its dynamic trafficking to the plasma membrane upon receptor stimulation and the identification of a direct binding partner, namely profilin-1. Because of profilin-1 binding, CLIC4 appears to function in a RhoA–mDia2–regulated actin polymerization network at the cell periphery, where it modulates integrin function, cell adhesion, and filopodia (10). Interestingly, we have identified conserved residues in CLIC4 whose equivalents mediate substrate binding in the GSTs and make up an elongated groove (cleft or concave surface) in CLIC4 that binds profilin-1 and are also essential for CLIC4 trafficking upon RhoA activation (15).

One challenge for future studies is to identify profilin-1 residues that are essential for binding to CLIC4 but do not perturb the interaction with poly-Pro–containing proteins and phosphatidylinositol 4,5-bisphosphate (29). Regardless of the precise CLIC4–profilin-1–binding mode, our data support a model in which RhoA–mDia2–driven actin polymerization triggers trafficking of the CLIC4–profilin-1 complex to the plasma membrane to integrate actin dynamics, integrin trafficking, and filopodium protrusion (Fig. 8, *B* and *C*). At the plasma membrane, CLIC4 harnesses filopodium length and density. Yet, CLIC4 does not affect formin-induced actin nucleation *in vitro* or actomyosin contractility. This suggests that CLIC4 acts by restricting tip protrusion. In this regard, both mDia2's processivity and polymerase activity are sensitive to profilin-1 (33). Thus, CLIC4 translocation could reduce either binding or activation of mDia2 by profilin-1. We hypothesize that CLIC4 may relay profilin-1- and tension-mediated regulation of formins (33), but more work is necessary to unravel the underlying mechanism.

CLIC4 has also been implicated in inhibiting the formation of branched actin on early endosomes through an unknown mechanism (13). Binding of CLIC4 to profilin-1 and the ability of profilin-1 to attenuate branched actin formation (40) may thus explain this puzzling observation. Profilin-1 is known to interact with multiple ligands and therefore is implicated in signaling processes beyond its actin-binding properties; furthermore, several studies have recently emerged that link mutations in *PFN1* to various human diseases (41, 42). Because some of these *PFN1* mutations are located in the poly-Pro–binding site, it will be interesting to examine their potential effect on CLIC4 function. Finally, future studies should assess whether the functions of other CLIC family members similarly rely on profilin-1 binding.

In conclusion, our findings provide new insights into the dynamic trafficking of CLIC4 to the plasma membrane upon RhoA activation by defining a new CLIC4-binding partner, namely the G-actin–binding protein profilin-1. Profilin-1 binding couples CLIC4 trafficking to RhoA–mDia2 signaling and filopodium formation. These results may have implications for filopodium-dependent processes, such as cell adhesion and the outgrowth of micrometastases (43).

## Experimental procedures

### Reagents and antibodies

1-Oleoyl-LPA, Y-27632, and SMIFH2 were from Sigma. EGF was from PeproTech. Type-I collagen was from Inamed BioMaterials. EDTA-free protease inhibitor mixture tablets were from Roche Applied Science. Phalloidin red (acti-stain^TM^555 phalloidin) was from Cytoskeleton, Inc. SMIFH2 was used as described previously (19). Antibodies used were as follows: monoclonal anti-β-actin (AC-15) and anti-FLAG M2 (Sigma); polyclonal anti-CLIC4, anti-mDia2, and anti-GFP, generated in-house (10, 21); monoclonal anti-mDia1 (D3) and polyclonal anti-profilin-1 (Santa Cruz Biotechnology); Alexa-Fluor–conjugated secondary antibodies (Invitrogen).

### Vectors

pGEX-6P1-mDia2 (FH1–FH2), pCDNA3 FLAG-mDia2 WT, pGEX-6P1-hPFN1, and pEGFP-C1-hPFN1 were previously described (18, 21, 34). pGEX-6P1-CLIC4 WT was previously described (10). pGEX-6P1-CLIC4(C35A) was generated from pGEX-6P1-CLIC4 WT using Phusion site-directed mutagenesis kit (ThermoFisher Scientific). For rescue experiments, 3×FLAG-tagged mouse CLIC4 WT (mCLIC4) and CLIC4(C35A) were cloned in LZRS-Blast vector. LZRS-IRES-GFP-RhoA(N19) and LZRS-IRES-GFP-RhoA(V14) were gifts from Dr. Jacques Neefjes.

### Cell Culture, infections, and transfections

HeLa cells were grown in Dulbecco's modified Eagle's medium (DMEM, Invitrogen) supplemented with 10% fetal bovine serum (FBS) under 5% CO_2_ at 37 °C. Stable HeLa CLIC4, mDia1, and mDia2 knockdown cells were previously described and characterized (10, 18, 44). For mDia2 and profilin-1 knockdown studies, four distinct shRNAs in the lentiviral vector pLKO.1 were employed (TRC human shRNA library; Sigma TRCN0000150903 (shmDia2 no. 1) and TRCN0000150850 (shmDia2 no. 2) (21, 44), TRCN0000311689 (shprofilin-1 no. 1), and TRCN0000294209 (shprofilin-1 no. 3)). pLKO.1 empty vector was used as control. Lentiviral production for the generation of knockdown cells was performed as described previously (10). Plasmid transfections for imaging studies were performed with X-tremeGene 9 (Roche Applied Science) reagent according to manufacturer's instructions.

### Live cell imaging, immunofluorescence, and image analysis

For live cell imaging experiments, cells were seeded on 24-mm glass coverslips and transiently transfected with YFP–CLIC4 WT or the C35A mutant. Cells were serum-starved overnight with DMEM supplemented with 0.1% FBS (starvation medium) and imaged in DMEM-F12 without phenol red (Invitrogen) under 5% CO_2_ at 37 °C on a Leica TCS-SP5 confocal microscope (×63 objective). We used minimal laser intensity to avoid photobleaching and phototoxicity. Series of confocal images were taken at 5 s. LPA was added 2 min after starting imaging. The translocation of YFP–CLIC4 was quantified using a home-built ImageJ analysis macro, essentially as described before (45); briefly, mean YFP intensity in membrane and cytosol in a time-lapse series of images was determined for the entire cell perimeter with the exclusion of any cell–cell contact region(s). Plasma membrane–cytosol (PM/Cyt.) ratios were normalized using the average PM/Cyt. ratio before stimulation as a reference. A similar approach was used to normalize plasma membrane and cytosolic CLIC4 signals. Confocal imaging of fixed cells was performed as described previously (10). Filopodium length at the basal membrane of phalloidin-stained cells was measured manually using ImageJ software.

### Super-resolution imaging

For super-resolution microscopy using the ground-state depletion imaging method (46), cells were cultured on 24-mm, no. 1.5 coverslips and transiently transfected with FLAG-mDia2. After 24 h, cells were serum-starved overnight, washed briefly with PBS, fixed with 4% paraformaldehyde for 10 min at room temperature, and permeabilized in cytoskeleton buffer supplemented with 0.1% Triton X-100 (47, 48). Samples were extensively washed with PBS and blocked with 5% BSA for 30 min at room temperature. Coverslips were incubated for 1 h with anti-FLAG M2 (Sigma) primary antibody at room temperature, washed, and incubated with Alexa-Fluor–conjugated secondary antibody (Alexa Fluor 647 and 488) and phalloidin (Alexa Fluor 488 and 647). Super-resolution microscopy was performed with a Leica SR GSD microscope (Leica Microsystems, Wetzlar, Germany) mounted on a Sumo Stage (no. 11888963) for drift-free imaging. Collection of images was done with an EMCCD Andor iXon camera (Andor Technology, Belfast, UK) and an oil immersion objective (HCX PL apo ×100, NA 1.47). Laser characteristics were 405 nm/30 mW, 488 nm/300 mW, and 647 nm/500 mW, with the 405-nm laser used for back pumping and the others for wide field/TIRF imaging. Ultra clean coverslips (cleaned and washed with base and acid overnight) were used for imaging. The number of recorded frames varied between 10,000 and 50,000, with a frame rate of 100 Hz. The data sets were analyzed with the Thunder Storm analysis module (49), and images were reconstructed with a detection threshold of 70 photons, sub pixel localization of molecules, and uncertainty correction, with a pixel size of 10 nm.

### Western blotting

Whole-cell lysates were prepared by lysing cells in JS lysis buffer (50 mm HEPES, pH 7.5, 150 mm NaCl, 1.5 mm MgCl_2_, 5 mm EGTA, 1% glycerol, 0.5% Triton X-100) supplemented with NaO_3_V_4_ (5 μm), NaF (1 μm), and protease inhibitor mixture (Roche Applied Science). Typically, 40 μg of total cell lysate were loaded on the gel. Membranes were blocked in nonfat dry milk and incubated with primary antibodies according to the manufacturers' instructions, followed by horseradish peroxidase-conjugated secondary antibodies (Dako Inc., 1:10,000).

### Densitometry

To estimate band intensities, nonsaturated Western blotting exposures were subjected to densitometric analyses using ImageJ.

### Recombinant proteins

GST-tagged CLIC4 (WT and C35A) was expressed in BL21 *Escherichia coli*, and CLIC4 proteins were purified using GSH-Sepharose ® 4B beads (GE Healthcare) and gel filtration. The GST tag was removed with PreScission Protease (GE Healthcare). Expression, purification, and cleavage of human profilin-1 were performed as described previously (18). Expression and purification of mDia2 (FH1–FH2 domain) were previously described (34), and cleavage was done as described for profilin-1 (18). Expression and purification of Rho–GTPase binding domains were previously described (50).

### FRET-based RhoA biosensor

The design and use of the FRET-based RhoA biosensor was described previously (17). Briefly, the complete amino acid sequence of a RhoA was positioned at the C terminus of a single polypeptide chain to preserve its interaction with guanine nucleotide dissociation inhibitor and other regulatory proteins. A FRET pair consisting of Cerulean3 and circularly permutated Venus was used. The HR1 region of PKN was used as the effector domain for activated RhoA. In a control biosensor, point mutation L59Q in PKN was introduced to generate a binding-deficient effector domain, so that FRET ratios remained at the basal level regardless of the activation state of RhoA.

### RhoA and Cdc42 pulldown assays

Rho–GTPase pulldown assays were performed as described previously (18, 34).

### Actin polymerization assays

Actin purification, bulk actin polymerization assays, and F-actin cosedimentation assays were performed as described previously (51, 52).

### Molecular modeling

We used the CLIC4 structure from PDB 2AHE and the profilin-1 structure from PDB 2PAV. For the HADDOCK modeling, we used the web interface in the expert mode (27). For CLIC4, we defined Phe-37, Pro-76, Asp-97, and Tyr-244 as “active” residues involved in binding. For profilin-1, we extracted all the residues that are in the surface but do not interact with actin in the 2PAV structure using AREIMOL from the CCP4 suite (53). 10,000 models were generated in HADDOCK, of which 500 were refined, and the best 200 were refined with water molecules and clustered. 196 out of the final water-refined 200 models clustered in four clusters with 132, 45, 10, and 9 models which, respectively, had HADDOCK scores of −122, −89, −90, and −87 and Z-scores of −1.7, 0.6, 0.5, and 0.7, respectively. The top cluster also showed a low internal RMSD (0.7 ± 0.5) and was clearly representing the most likely model for the interaction. Details of the HADDOCK scoring are shown in Table S1. Molecular structures in Fig. 4 were prepared by CCP4MG (54).

### SPR

SPR experiments were carried out on a Biacore T200 machine (GE Healthcare) at 25 °C. A GST antibody from the GST capture kit (GE Healthcare) was covalently bound on a CM5 sensor chip via amino coupling. GST-tagged CLIC4 was immobilized on the experiment flow cell, and the control flow cell had an equal amount of GST. A concentration series of human profilin-1 was injected over the chip surface using running buffer (20 mm HEPES/NaOH, pH 7.5, 100 mm NaCl, 1 mm tris(2-carboxyethyl)phosphine, 0.05% v/v Tween 20, and 1 mg/ml BSA). The equilibrium dissociation constant (*K_D_*) of the CLIC4–profilin-1 complex was determined by plotting steady-state equilibrium values against profilin-1 concentration and fitting these data to a single site-binding model using Graphpad Prism 7.

### Quantification of filopodial metrics

Filopodium length was measured manually using ImageJ because FiloQuant plugin (55) often underestimated long filopodia because of incomplete tracing or fragmentation. Filopodium density (ratio between the number of filopodia and the total cell perimeter in a given image) was obtained using FiloQuant (55).

### Imaging, tracking, validation, and analysis of filopodial dynamics

Control KD and CLIC4 KD cells were plated on collagen-coated coverslips and, 1 day later, starved overnight in DMEM supplemented with 0.1% FCS. The next day, Sir-Actin (Cytoskeleton Inc., 0.5 μm) was added 1 h prior to imaging. Next, coverslips were transferred in a metal O-ring (Invitrogen) and overlaid with Ham's F-12 imaging medium (Gibco) devoid of FCS. Cells were filmed with an SP8 confocal microscope (Leica) equipped with an environmental control chamber set at 37 °C at 5% CO_2_. Twenty 1024 × 1024 pixel frames/min were acquired with line accumulation set on 3 by using a ×40 water-immersion objective (1.10 N.A.) and a ×2.5 digital zoom. After ∼5 min of imaging, LPA (5 μm) was added.

Two independent movies were acquired for each cell line. Movies were processed and analyzed using ImageJ as follows: time series were converted to a 512 × 512 pixel format, bleach corrected, and registered with the ImageJ plugin StackReg. To prevent pipetting artifacts, frames encompassing addition and resuspension of LPA (typically 3–4) were removed prior to initiating filopodial tracking, which was done manually using ROI manager. As filopodia are usually dimmer than cell bodies, image brightness was adjusted manually. Only filopodia visible both prior to and after LPA addition were selected for tracking. Traces were processed with FilamentDetector plugin for computer-aided identification of the plus (more dynamic) tip. Only traces with identified plus tip located distally from the cell body were included in all subsequent analyses. Filopodial length was normalized to the value measured at the time of LPA addition wherever normalized filopodium length is depicted.

### RT-qPCR

*PFN1* expression analysis was performed as described previously (21). Validated *PFN1* primers were as follows: forward, GGGTGGAACGCCTACATCG, and reverse, CCATTCACGTAAAAACTTGACCG.

### Statistical analysis

For determination of statistical significance, unpaired two-tail Student's *t* test, one-way ANOVA, and segmental linear regression were performed using GraphPad Prism 7 software. Significance values are compared with either control conditions or among each other.

## Author contributions

E. A., W. H. M., and M. I. conceptualization; E. A., T. I., W. H. M., and M. I. resources; E. A., K. M. K., L. N., A. P., W. H. M., and M. I. formal analysis; E. A. validation; E. A., J. K., T. I., A. P., W. H. M., and M. I. investigation; E. A., J. K., K. M. K., L. N., A. P., K. J., W. H. M., and M. I. methodology; E. A., W. H. M., and M. I. writing-original draft; E. A., W. H. M., and M. I. writing-review and editing; K. M. K. visualization; A. P. software; K. J., W. H. M., and M. I. supervision; W. H. M. funding acquisition.

## Supplementary Material

Supporting Information

## Footnotes

3 The abbreviations used are:

CLIC: chloride intracellular channel
GST: glutathione *S*-transferase
IRES: internal ribosome entry site
LPA: lysophosphatidic acid
PDB: Protein Data Bank
ANOVA: analysis of variance
DAPI: 4′,6-diamidino-2-phenylindole
EGF: epidermal growth factor
EGFR: EGF receptor
ROCK: Rho-kinase
DMEM: Dulbecco's modified Eagle's medium
FBS: fetal bovine serum
FCS: fetal calf serum
PM/Cyt.: plasma membrane/cytosol ratio
mW: milliwatt
RMSD: root mean square deviation
SPR: surface plasmon resonance.
