# Supplementary material for: Profilin binding couples chloride intracellular channel protein CLIC4 to RhoA–mDia2 signaling and filopodium formation
Source: J Biol Chem. 2018 Oct 31;293(50):19161–76. doi: 10.1074/jbc.RA118.002779 (PMC6302171; doi:10.1074/jbc.RA118.002779)
Supplement: Supporting Information [file supp_293_50_19161__index.html]

Profilin 1 binding couples chloride intracellular channel protein CLIC4 to RhoA–mDia2 signaling and filopodium formation — CLIC4 as a profilin-binding protein — Profilin binding couples chloride intracellular channel protein CLIC4 to RhoA–mDia2 signaling and filopodium formation — CLIC4 as a profilin-binding protein — Supporting Information 

# Profilin binding couples chloride intracellular channel protein CLIC4 to RhoA–mDia2 signaling and filopodium formation

## Supporting Information

- Supporting Information - Supporting Table S1, Figs. S1-S8 and Legends to Movies S1-S4.
- Movie S1 - CLIC4 trafficking upon LPA addition.
- Movie S2 - CLIC4 trafficking upon EGF addition
- Movie S3 - Filopdial dynamics in HeLa cells
- Movie S4 - Filopodial dynamics in CLIC4 knockdown eLa cells
