# Supplementary material for: Profilin binding couples chloride intracellular channel protein CLIC4 to RhoA–mDia2 signaling and filopodium formation
Source: J Biol Chem. 2018 Oct 31;293(50):19161–76. doi: 10.1074/jbc.RA118.002779 (PMC6302171; doi:10.1074/jbc.RA118.002779)
Supplement: Supporting Information [file supp_RA118.002779_136503_3_supp_226791_phxxvq.pdf]

Profilin 1 binding couples chloride intracellular channel protein CLIC4 to RhoA–mDia2 signaling and filopodium formation

Elisabetta Argenzio[1], Jeffrey Klarenbeek[1], Katarzyna M. Kedziora[1], Leila Nahidiazar[1], Tadamoto Isogai[2], Anastassis Perrakis[3], Kees Jalink[1], Wouter H. Moolenaar[1,*], and Metello Innocenti[2,*]

Supporting Information

List of the material included:
- One Supporting Table (Table S1), eight Supporting Figures (Figure S1-S8) and Supporting Movie legends.

TABLE S1. HADDOCK modeling parameters

	Cluster 1	Cluster 2	Cluster 3	Cluster 4
**HADDOCK score**	-121.8 ± 2.8	-89. ±2.0	-90.8 ±6.7	-87.0 ± 4.5
**Cluster size**	132	45	10	9
**RMSD**	0.7 ± 0.5	13.0 ± 0.0	9.9 ± 0.2	9.2 ±0.6
**Van der Waals energy**	-58.9 ± 5.2	-49.3 ± 8.3	-52.4 ± 6.1	-52.0 +/- 5.2
**Electrostatic energy**	-291.5 ±54.1	-228.3 ± 51.2	-226.6 ± 24.8	-177.9 ± 33.8
**Desolvation energy**	-4.7 ± 6.2	5.5 ± 9.9	6.8 ± 12.1	0.4 +/- 6.5
**Restraints violation e.**	1.2 ± 0.78	2.7 ± 3.12	1.3 ± 0.79	0.8 +/- 0.40
**Buried Surface Area**	1979.0 ± 65.8	1702.0 ± 105.3	1613.1 ± 82.5	1656.7 ± 58.3
**Z-Score**	-1.7	0.6	0.5	1656.7 ± 58.3

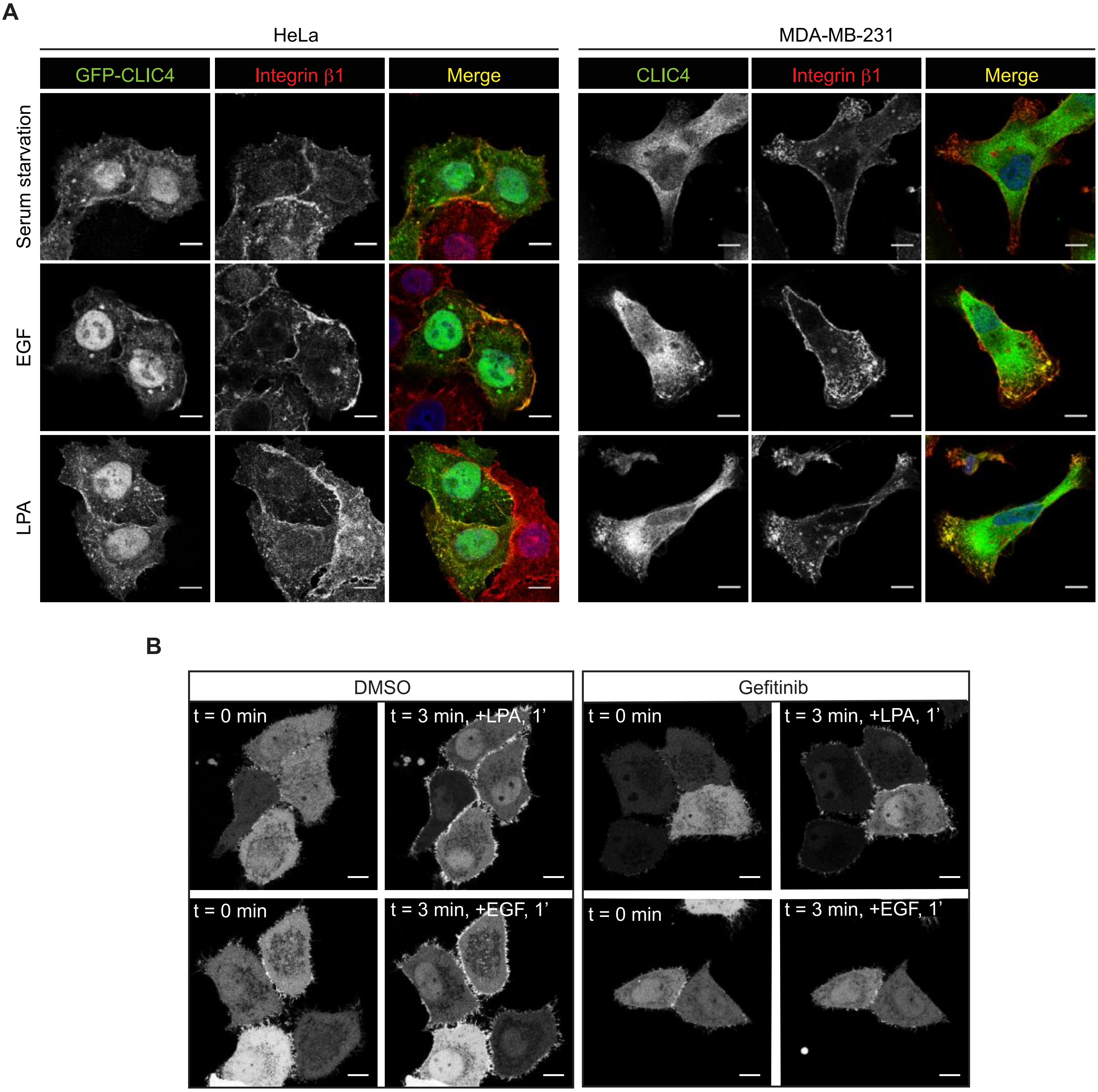

Figure S1. EGF- and LPA-induced translocation of CLIC4 to the plasma membrane in Hela and MDA-MB-231 cells.
(A) CLIC4 colocalizes with integrin β1 upon both LPA and EGF stimulation. HeLa and MDA-MB-231 cells were seeded on collagen-I-coated coverslips, transfected with GFP-CLIC4 (Hela cells), serum starved overnight and stimulated with either LPA (2 μM) or EGF (100 ng/nl) for 2 minutes. Representative confocal sections show GFP-CLIC4 in HeLa cells or endogenous CLIC4 in MDA-MB-231 cells (green in merge), integrin β1 (red in merge) and DAPI (blue in merge). Scale bars: 10 μm. **(B)** LPA does not act through EGFR transactivation. HeLa cells on collagen-I-coated coverslips, transfected with YFP-CLIC4 and serum-starved overnight, were treated with DMSO or EGFR inhibitor Gefinitib (2 μM) during 30 minutes before stimulation. Frames from time-lapse movies at the indicated time points are shown. Scale bars: 10 μm.

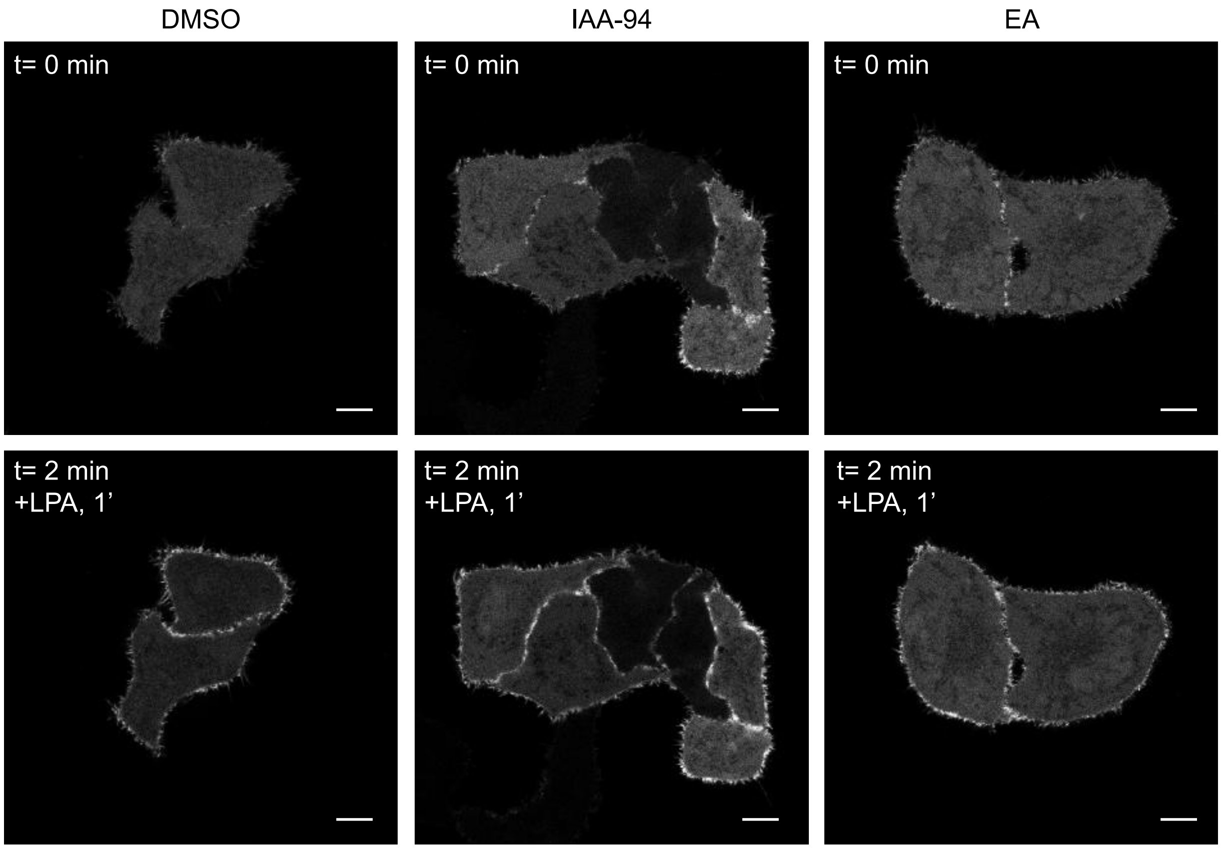

Figure S2. IAA-94 and ethacrynic acid do not inhibit CLIC4 traslocation induced by LPA.
Control knockdown Hela cells on glass coverslips were transfected with YFP-CLIC4. Serum-starved cells were incubated with IAA-94 (5 µM) or ethacrynic acid (EA, 5 µM) for 30 minutes before LPA stimulation. DMSO treatment served as a control. Frames from time-lapse movies at the indicated time points are shown. Scale bars: 10 µm.

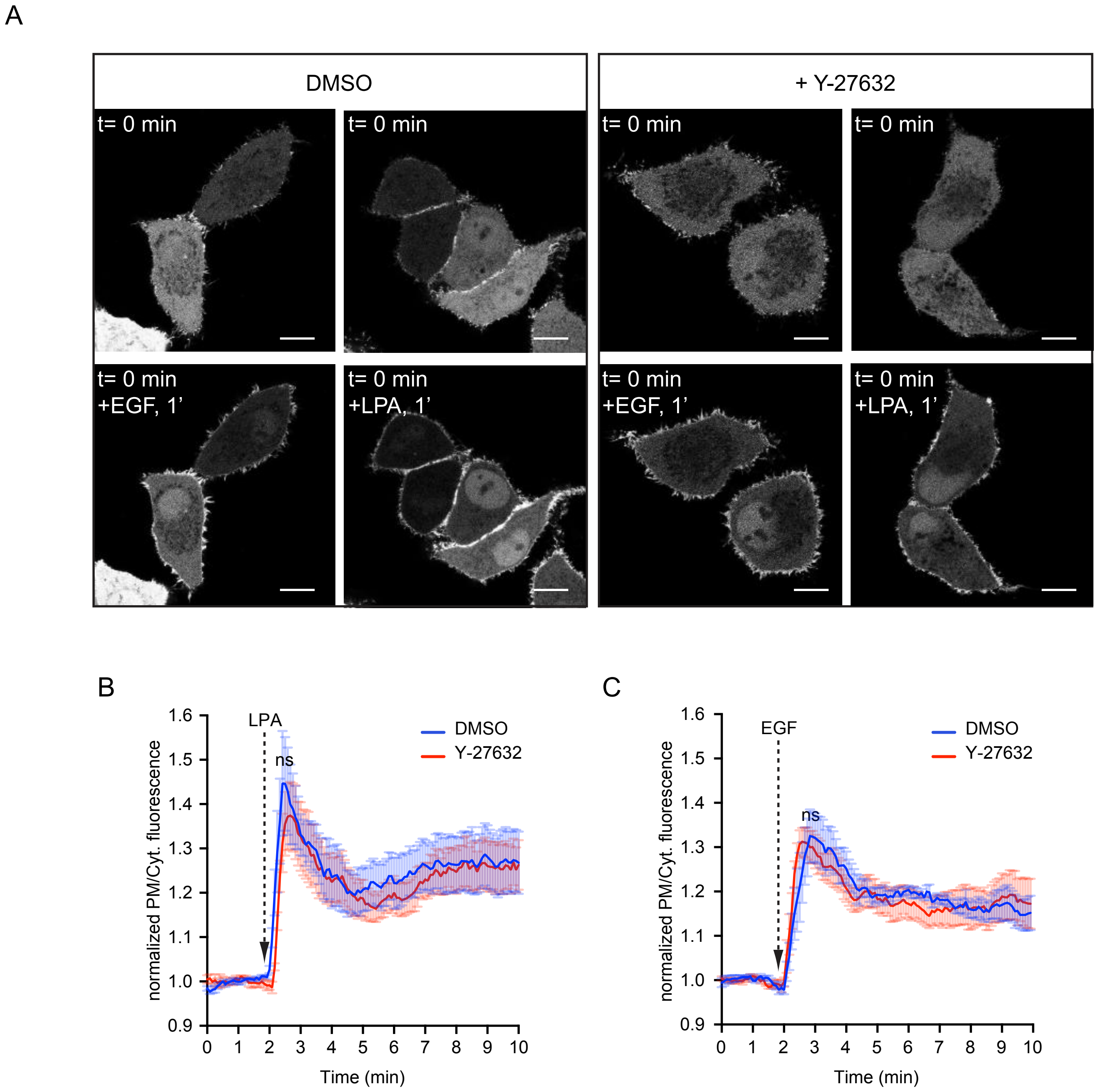

Figure S3. CLIC4 translocation is independent of ROCK activity.
(A) Live-cell imaging of CLIC4 translocation in HeLa cells. Control knockdown cells on glass coverslips were transfected with YFP-CLIC4. Serum-starved cells were treated with either DMSO or Y-27632 (10 µM, 30 min.) and LPA (2 µM) or EGF (100 ng/ml) was added at 2 minutes after starting imaging. Frames from time-lapse movies at the indicated time points are shown. Scale bars: 20 µm. **(B,C)** Quantification of LPA- and EGF-induced CLIC4 translocation (LPA and EGF: DMSO = 5 cells, Y-27632 = 4 cells). Unpaired two-tailed t test was performed comparing the highest values of the curves (ns, non-significant). Net translocation is expressed as mean ± s.e.m. of the normalized PM/Cyt. ratio obtained from the analyzed cells.

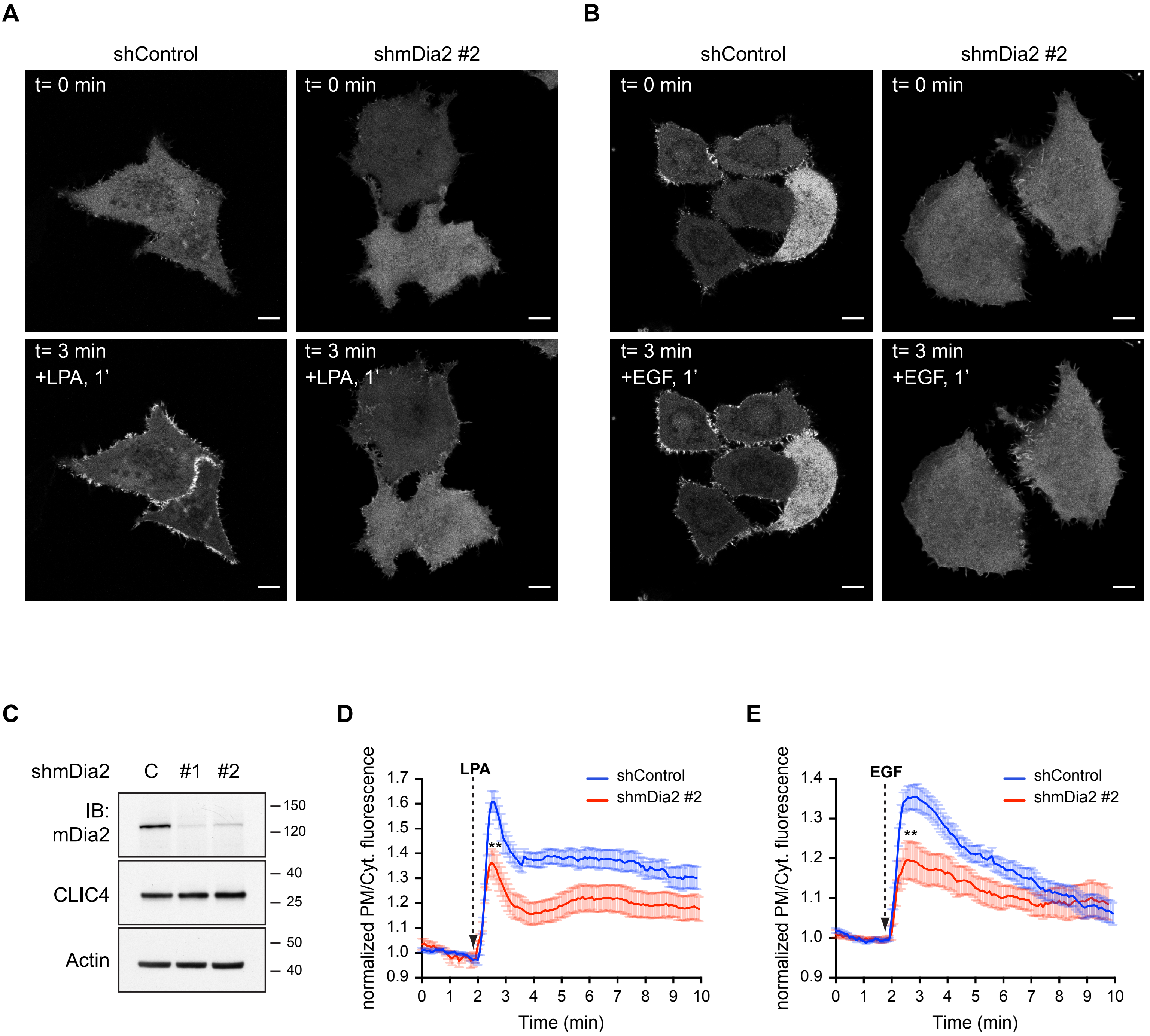

Figure S4. CLIC4 translocation depends on mDia2.
(A,B) Live-cell imaging of CLIC4 translocation in mDia2 knockdown HeLa cells. A second stable population of mDia2 knockdown cells was obtained as described in the Experimental procedures using hairpin shmDia2 #2. Control (shControl) and mDia2 (shmDia2 #2) knockdown cells were seeded on glass coverslips, transfected with YFP-CLIC4 and serum starved overnight. LPA (2 µM) in (A) and EGF (100 ng/ml) in (B) were added 2 minutes after starting imaging. Frames from time-lapse movies at the indicated time points are shown. Scale bars: 10 µm. **(C)** mDia2 knockdown efficiency. Total cell lysates obtained from control (C), mDia2 #1 and mDia2 #2 (#1 and #2) knockdown cells were immunoblotted (IB) using anti-mDia2 antibody to determine mDia2 expression. β-actin (Actin) was used as loading control. Hairpin #1 and #2 reduced mDia2 protein levels by 96±1% and 91±3% (mean ± s.e.m., n= 3). **(D,E)** LPA- and EGF-induced CLIC4 translocation (LPA: $n_{shControl}$= 14 cells, $n_{shmDia2\ \#2}$= 14 cells, from two independent experiments; EGF: $n_{shControl}$= 20 cells, $n_{shmDia2\ \#2}$= 12 cells, from two independent experiments). Net translocation is expressed as mean ± s.e.m. of the normalized PM/Cyt. ratio obtained from the analyzed cells. Unpaired two-tailed t tests were performed comparing the highest values of the curves (**p<0.01).

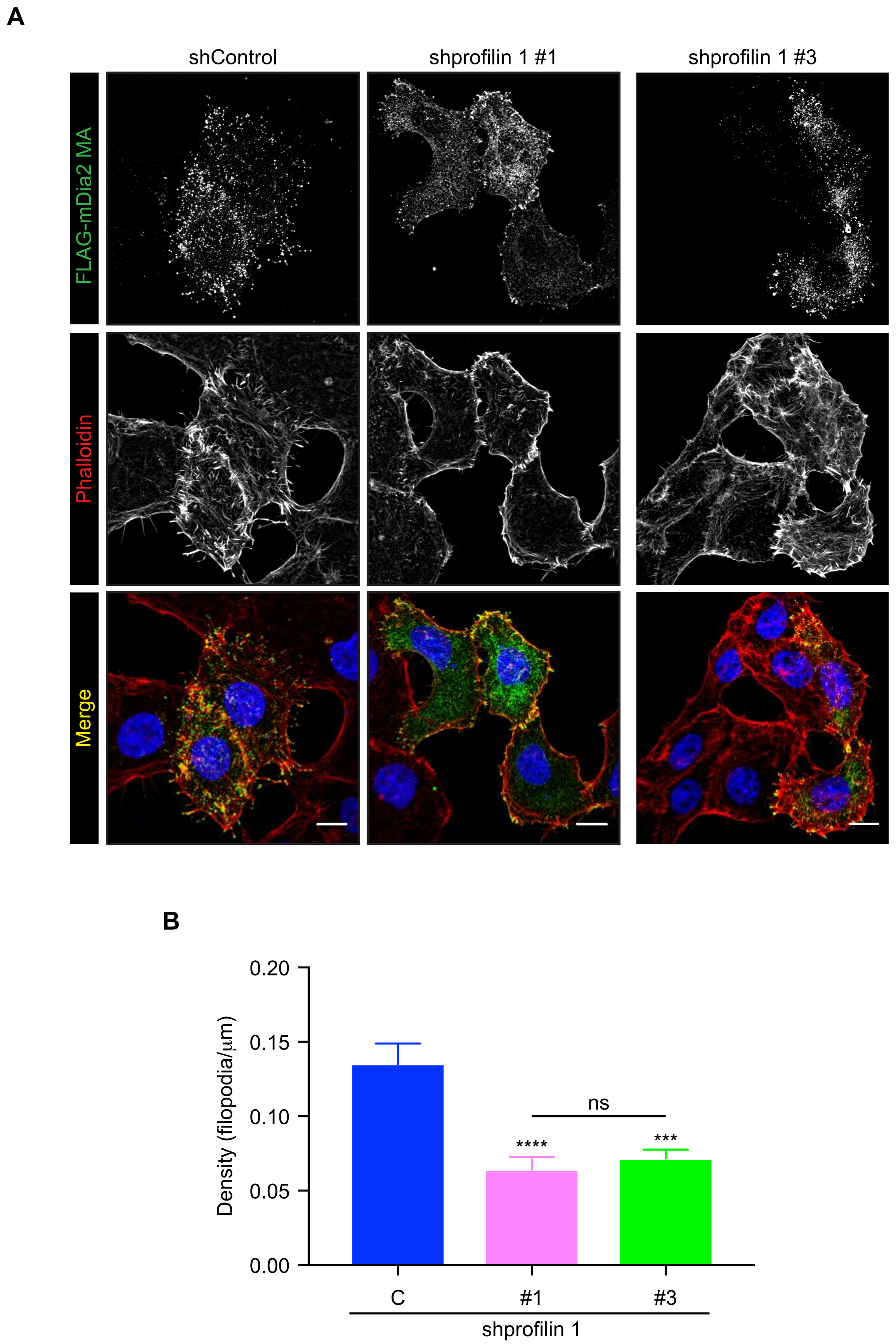

Figure S5. Filopodium formation by mDia2 is impaired upon profilin 1 depletion.
(A) Control (shControl) and profilin-1 (shprofilin 1 #1 and shprofilin 1 #3) knockdown HeLa cells seeded on collagen-I-coated glass coverslips were transfected with constitutively active mDia2 (FLAG-mDia2 MA). Cells were serum starved overnight, fixed and stained. Representative maximal projections of confocal Z-stacks show phalloidin (red), Flag-mDia2 MA (green) and DAPI (blue). Scale bars, 10 µm. **(B)** Density of mDia2-induced filopodia is reduced upon Profilin-1 knockdown. Bar graph depicts mean ± s.e.m. of filopodial density from the analyzed images ($n_{shControl}$= 7, $n_{shprofilin\ 1\ \#1}$= 7, $n_{shprofilin\ 1\ \#3}$= 8, from three independent experiments). One-way ANOVA with Tukey's multiple comparisons test was performed (***p<0.001, ****p<0.0001, ns, non-significant).

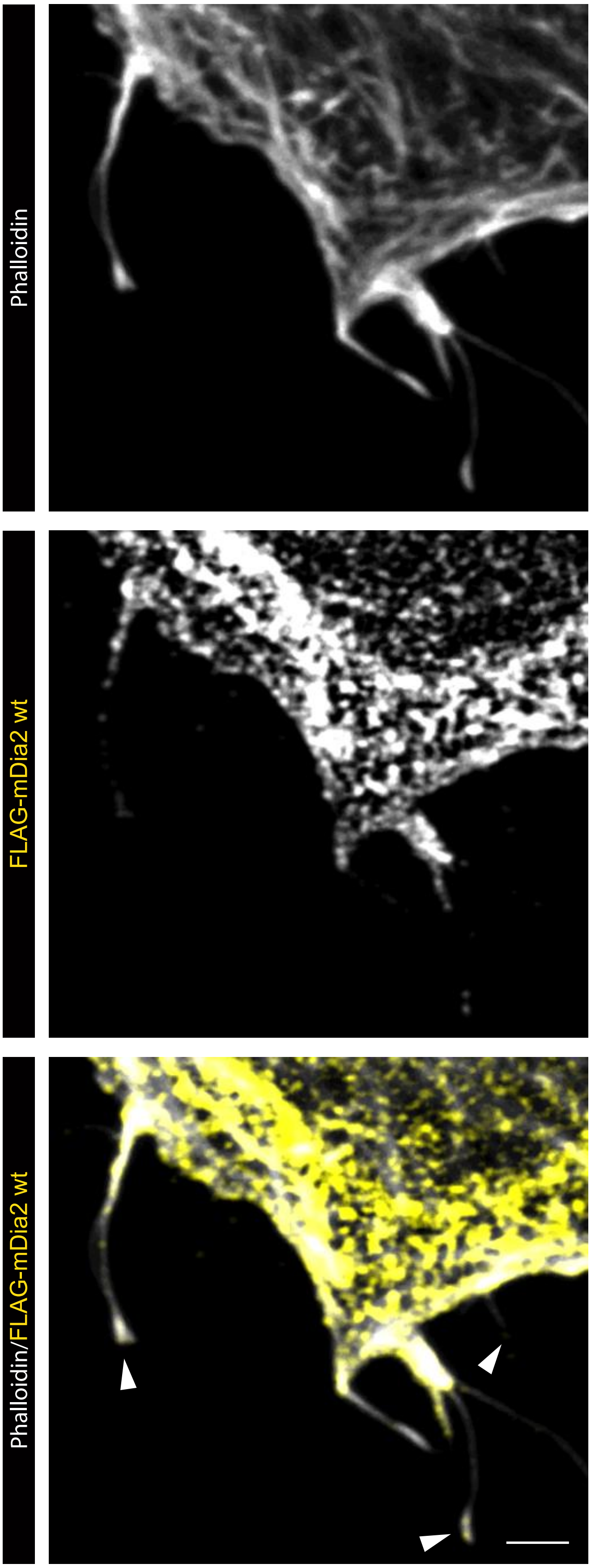

Figure S6. mDia2 localizes at the tip of filopodia in CLIC4 KD cells.
CLIC4 knockdown (shCLIC4 #3) HeLa cells were seeded on collagen-I-coated coverslips, transfected with FLAG-mDia2 wt and serum starved overnight. Cells were fixed and stained with anti Flag antibody (yellow) and phalloidin (grey) to detect mDia2 and the actin cytoskeleton, respectively. Images depict a representative confocal basal section. Arrowheads mark filopodial tips showing mDia2 enrichment in the merge. Scale bar: 2 µm.

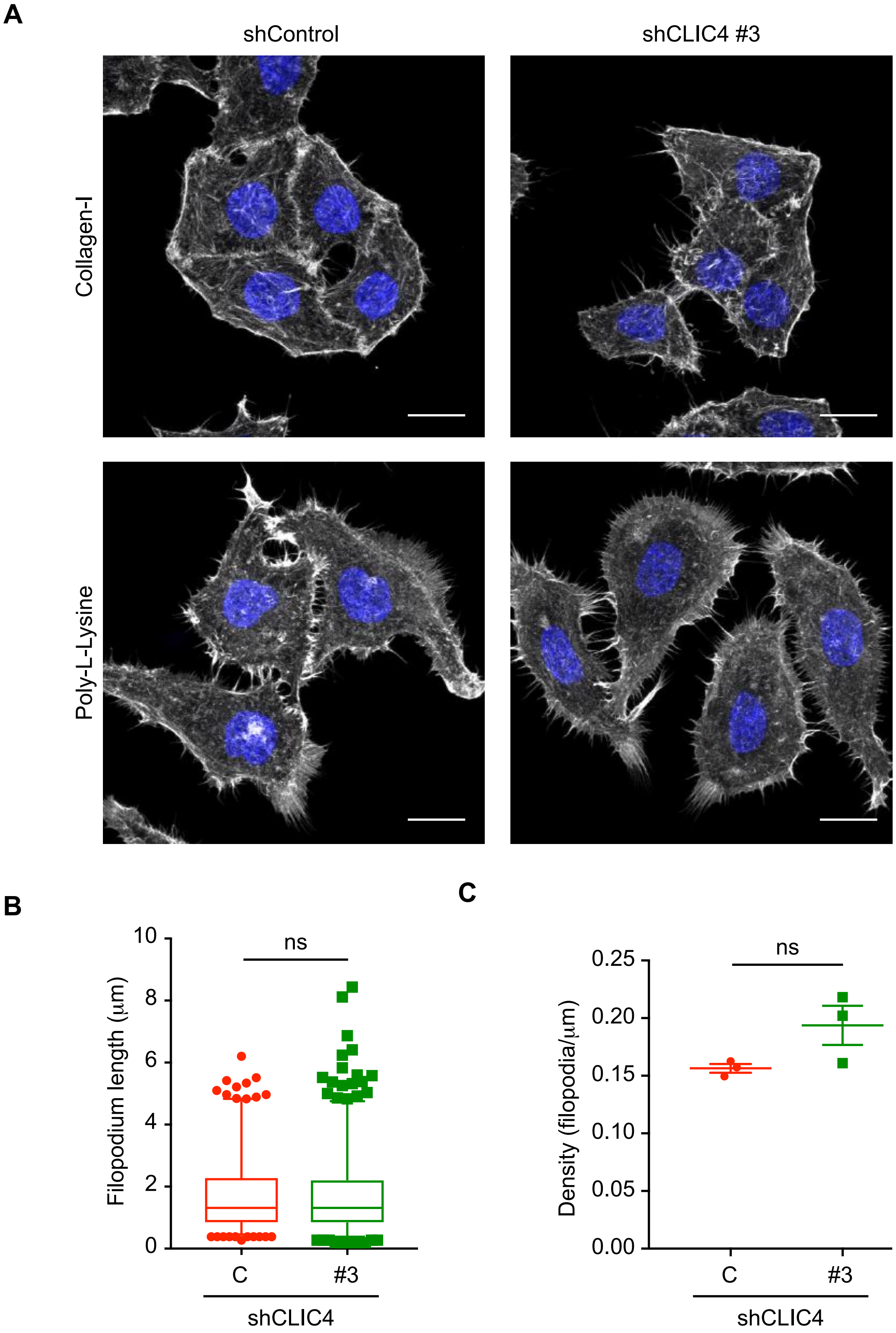

Figure S7. CLIC4 is involved in regulating integrin-dependent filopodia.
(A) Cell morphology on collagen-I or poly-Lysine. Control (shControl) and CLIC4 knockdown (shCLIC4 #3) HeLa cells were seeded on coverslips coated with the indicated substrates and serum-starved overnight. Maximal projections of confocal Z-stacks show actin cytoskeleton and nuclei stained with phalloidin (grey) and DAPI (blue), respectively. Scale bars, 10 µm. **(B,C)** Quantification of filopodium length and density on poly-L-Lysine. Filopodium length in (B) was measured using FiloQuant. Boxes represent the 25th to 75th percentiles and whiskers indicate the median and the 5th and the 95th percentiles, respectively. Filopodium density (C) is expressed as mean ± s.e.m. ($n_{shControl}$= 3 images, $n_{shCLIC4\ \#3}$= 3 images). Unpaired two-tailed t tests were performed (ns, non-significant).

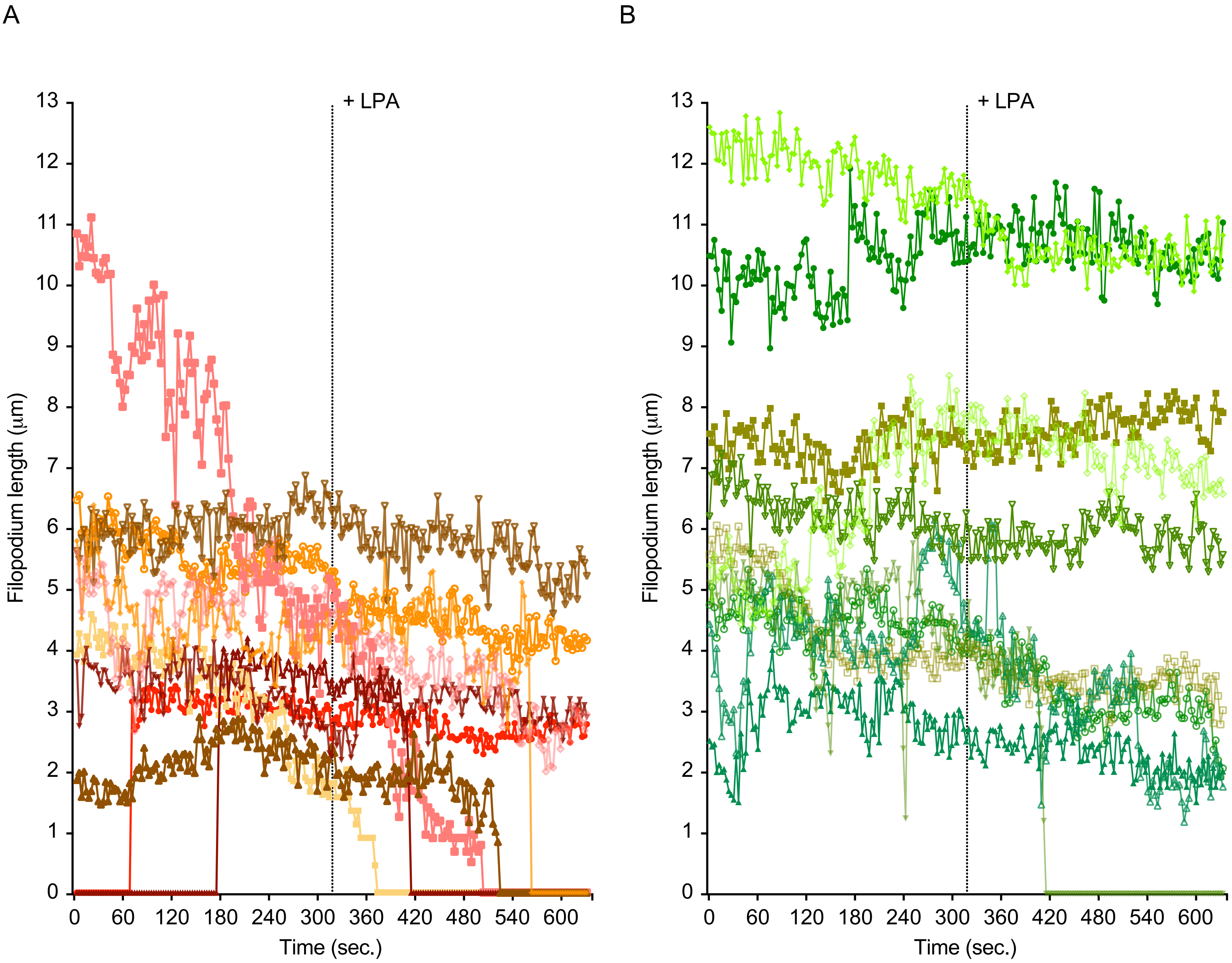

Figure S8. Filopodial dynamics before and after LPA stimulation.
(A) Control (shControl) HeLa cells were seeded on collagen-I-coated glass-bottom Petri dishes and serum starved overnight. Subsequently, cells were labeled with Sir-Actin and imaged as described in the Experimental procedures. Graph illustrates how the length (measured in µm) of ten basal filopodia obtained from eight different cells and two independent experiments changes over time (sec. = seconds). LPA addition (+ LPA) is indicated by vertical dotted line. **(B)** CLIC4 knockdown (shCLIC4 #3) HeLa cells were seeded on collagen-I-coated glass-bottom Petri dishes and serum starved overnight. Subsequently, cells were labeled and imaged as above. Graph illustrates how the length (measured in µm) of ten basal filopodia obtained from seven different cells and two independent experiments changes over time (sec. = seconds). LPA addition (+ LPA) is indicated by vertical dotted line.

Supporting Movie legends

Movie 1. CLIC4 translocation induced by LPA in HeLa cells.
Note the transient character of CLIC4 accumulation at the plasma membrane, concomitant with homogenous depletion of cytosolic GFP-CLIC4 upon addition of LPA (2 µM). Images were collected every 10 seconds and shown at 5 frames per second.

Movie 2. CLIC4 translocation induced by EGF in HeLa cells.
Experimental conditions as in Movie 1. EGF was added at 100 ng/ml.

Movie 3. Filopodial dynamics in control knockdown HeLa cells.
Cells were seeded on collagen-I-coated glass-bottom Petri dishes, serum starved overnight and then labeled with Sir-Actin. *Left,* actin cytoskeleton. *Right,* tracked filopodia are shown as white lines with white empty circles indicating the position of dynamic plus tips. Images were collected every 3 seconds and are displayed at 18 frames per second. Addition of LPA (5 µM) is marked as + LPA.

Movie 4. Filopodial dynamics in CLIC4 knockdown HeLa cells.
Cells were seeded on collagen-I-coated glass-bottom Petri dishes, serum starved overnight and then labeled with Sir-Actin. *Left,* actin cytoskeleton. *Right,* tracked filopodia are shown as white lines with white empty circles indicating the position of dynamic plus tips. Images were collected every 3 seconds and are displayed at 18 frames per second. Addition of LPA (5 µM) is marked as + LPA.
